# Photothermal Chemistry Based on Solar Energy: From Synergistic Effects to Practical Applications

**DOI:** 10.1002/advs.202103926

**Published:** 2021-11-26

**Authors:** Jianan Hong, Chenyu Xu, Bowen Deng, Yuan Gao, Xuan Zhu, Xuhan Zhang, Yanwei Zhang

**Affiliations:** ^1^ State Key Laboratory of Clean Energy Utilization Zhejiang University Hangzhou 310027 China; ^2^ Department of Chemical and Materials Engineering University of Alberta Edmonton Alberta T6G 1H9 Canada; ^3^ Graduate School of Chemical Sciences and Engineering Hokkaido University Sapporo 060‐0814 Japan

**Keywords:** full spectrum, photothermal, solar energy

## Abstract

With the development of society, energy shortage and environmental problems have become more and more outstanding. Solar energy is a clean and sustainable energy resource, potentially driving energy conversion and environmental remediation reactions. Thus, solar‐driven chemistry is an attractive way to solve the two problems. Photothermal chemistry (PTC) is developed to achieve full‐spectral utilization of the solar radiation and drive chemical reactions more efficiently under relatively mild conditions. In this review, the mechanisms of PTC are summarized from the aspects of thermal and non‐thermal effects, and then the interaction and synergy between these two effects are sorted out. In this paper, distinguishing and quantifying these two effects is discussed to understand PTC processes better and to design PTC catalysts more methodically. However, PTC is still a little far away from practical. Herein, several key points, which must be considered when pushing ahead with the engineering application of PTC, are proposed, along with some workable suggestions on the practical application. This review provides a unique perspective on PTC, focusing on the synergistic effects and pointing out a possible direction for practical application.

## Graphical abstract

Solar‐driven chemistry is an attractive way to solve energy and environmental problems, among which photothermal chemistry (PTC) can realize full‐spectral utilization of solar radiation and driving chemical reactions more efficiently under relatively mild conditions. In order to push ahead with the engineering application of PTC, multisystem integration can be considered.

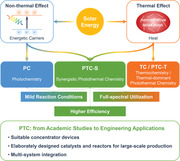

## 1 Introduction

With the growing population and the rapid development of the global economy, energy shortage and environmental deterioration have become two major issues threatening the survival and development of humans. Solar energy, which is clean, sustainable and sufficient to fulfill the global energy demand, is considered as a promising alternative to fossil fuels.^[^ ^1^ ^]^ To date, a variety of ways to utilize solar energy have been developed. Among them, using solar irradiation to drive chemical reactions is an attractive way to solve both energy and environmental problems.^[^ ^2^, ^3^ ^]^ Storing solar energy in chemical bonds makes the utilization of solar energy less affected by its discontinuity and instability, which can also match well with existing energy systems.^[^ ^4^, ^5^ ^]^ Solar energy can also be the driving force for environmental remediations, such as water treatment, air purification and disinfection, removing substances that are harmful to the environment or to the human body.^[^ ^6^, ^7^ ^]^ Energy and environmental tasks can even be fulfilled in one process. For example, using solar energy to produce valuable solar fuels from CO_2_ can not only meet a part of the energy demand, but also reduce CO_2_ emission.^[^ ^8^ ^]^

**Figure 1:**
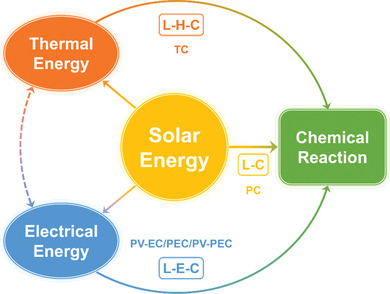
The main strategies to drive chemical reaction by solar energy: L–C (Light–Chemistry), L–E–C (Light–Electricity–Chemistry), and L–H–C (Light–Heat–Chemistry). PC: photochemistry; TC: thermochemistry; PV‐EC: photovoltaic electrochemistry; PEC: photoelectrochemistry; PV‐PEC: photovoltage‐assisted photoelectrochemistry.

To drive chemical reactions by solar energy, there are three main strategies considering energy conversion processes, as shown in **Figure** **1**.

The most direct strategy is photochemistry (PC), which takes place under effective light irradiation and usually needs a photoactive catalyst to produce photoinduced carriers with high energy to activate the reactants. We label this strategy as Light–Chemistry (L–C). PC has the advantage of mild reaction conditions, and the photoactive catalyst is typically a semiconductor or a kind of composite material containing at least one semiconductor, whose energy bandgap allows it to absorb and utilize only photons with higher energy than the bandgap. Most semiconductors used for catalysis can only utilize very little of the solar spectrum. Although many efforts have been made to expand the light absorption of semiconductor catalysts into the visible region, the efficiency is still limited by the poor separation and migration of photoinduced carriers.^[^ ^9^ ^]^ Moreover, the mild reaction conditions can be an advantage but also a drawback, which impedes the large‐scale application of PC due to the sluggish reaction dynamics.

Solar energy can be converted into electrical energy before driving chemical reactions, and this strategy is labeled as Light–Electricity–Chemistry (L–E–C). There are several types of systems that follow this strategy: photovoltaic electrochemistry (PV‐EC), photoelectrochemistry (PEC), and photovoltage‐assisted photoelectrochemistry (PV‐PEC). PV‐EC first generates electrical energy from sunlight, and then drives chemical reactions by means of electrochemistry. PEC can be considered as the integration of a PV cell and electrodes or be regarded as the separation of the PC process into two half reactions. While the required electrical potential bias for PEC is provided by a PV cell in PV‐PEC.^[^ ^10^ ^]^ Compared with the L–C strategy, electrons can be collected and separated by wires in the L–E–C strategy, which makes the efficiency of photoinduced electrons much higher.^[^ ^11^ ^]^ However, the L–E–C strategy is also limited by the spectral absorptivity. The PV cells or the electrodes of PEC systems require energetic photons to overcome the bandgaps, while long‐wavelength photons are useless or even detrimental to the solar‐to‐electricity efficiencies because of the elevated temperature.

For solar fuel production or other uphill energy conversion processes, the absorption of the solar spectrum determines the maximum energy efficiency. Without the assistance of any other energy source, including chemical energy (e.g., sacrificial agent) and electrical energy (e.g., biased voltage), the highest experimental solar‐to‐fuel (STF) efficiency is only several percent for PC and PEC. Although higher efficiency can be attained in PV‐EC systems, it is still less than 20%.^[^ ^11^ ^]^

In addition to electrical energy, solar energy can also be initially converted into thermal energy for thermochemistry (TC), which we term it as Light–Heat–Chemistry (L–H–C). To achieve the temperature required by the chemical reactions, materials with excellent light absorption, concentrating devices, insulation structures, and thermal management are all necessary. Unlike the L–E–C strategy, L–H–C can make use of the entire spectrum. With appropriate materials and ingenious structural designs, the efficiencies of light‐to‐heat conversion can reach nearly 100%.^[^ ^12^ ^]^ However, the reaction conditions remain tough, sometimes requiring high temperature and high pressure to obtain appreciable results, and the heating of the reaction system to high temperature results in irreversibility. The highest STF efficiency of the solar thermochemical cycle for H_2_O splitting and CO_2_ reduction is only ≈5% attained in the experimental validation.^[^ ^13^ ^]^ According to the second law of thermodynamics, heat generation will increase irreversible losses and reduce the quality of energy. Considering the potential of high‐energy photons, it seems somewhat “wasteful” to use the whole solar spectrum for heating.

**Figure 2:**
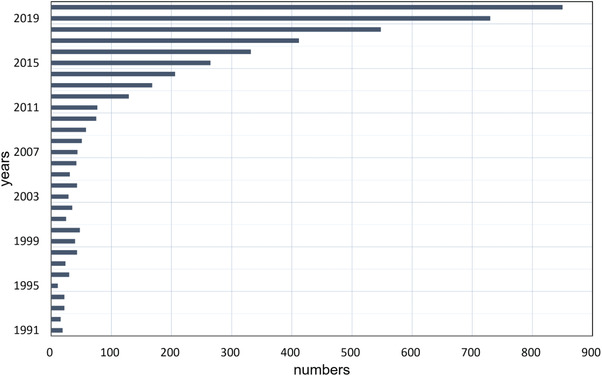
Numbers of researches about PTC in the past 30 years (searched by topic: (phototherm* OR phototherm* OR thermophoto* OR thermophoto*) AND topic: (cataly* OR chemi*) at Web of Science Core Collection).

It is clear that there are some limitations in a single process. L–C has drawbacks in terms of spectral efficiency and reaction rate, while L–H–C wins the quantitative game but loses the qualitative game. Some researchers have begun to consider a combination of L–C and L–H–C strategies, intending to achieve full‐spectral cascade utilization of solar radiation and to drive chemical reactions more efficiently under relatively mild conditions. This hybrid strategy is commonly known as “photothermal chemistry” or “photo‐thermochemistry” (PTC), where thermal and non‐thermal effects may work together in chemical reactions. The number of studies on PTC has been increasing year by year, especially in the last decade, as shown in **Figure** **2**. Focusing on a variety of materials and applications, the concept of coupling thermal and non‐thermal effects for chemical reactions has been realized and some PTC mechanisms have been explored. However, quite a few of PTC researches are at a laboratorial stage with small scales and low yields, and there is still an enormous gap between academic studies and engineering applications. With the increasing attention to PTC in recent years, several reviews relevant to PTC from different perspectives have been published. Most reviews introduce the latest progress and propose research prospects, mainly focusing on a certain field of applications or a certain type of materials.^[^ ^2^, ^10^, ^14^, ^15^, ^16^, ^17^ ^]^ Some introduce the mechanisms, materials and applications in a larger scope.^[^ ^3^, ^18^ ^]^ In this review, we focus on the synergistic effects in PTC and the development from academic studies to engineering applications, hoping to discuss the practice of PTC from a general perspective. We first introduce the basic mechanisms of PTC, summarizing the interaction and synergy of thermal and non‐thermal effects in PTC processes. Then, strategies and challenges to distinguish and quantify these two effects are discussed, which is necessary to understand the PTC processes, guide the design of PTC catalysts, and optimize the PTC reaction conditions. Later, considering that the ultimate aim of PTC academic studies is to move toward practical applications, we propose several key points which must be considered when pushing ahead with the engineering application of PTC, expecting to provide some workable suggestions for practical applications.

## 2 Comprehending of PTC with Synergistic Effects

Although “photothermal” has attracted increasing attention in the field of chemical catalysis in recent years, and is far from rare in biomedical applications and water vaporization, it is still a confusing item.^[^ ^19^, ^20^, ^21^ ^]^ In biomedical applications or water vaporization processes, “photothermal” generally refers to the conversion of incident light into thermal energy by certain materials or structures. However, when the “photothermal” nature of PTC is discussed, it cannot be as simple as “light‐to‐heat conversion.” On the whole, PTC can be mainly divided into two types according to the effect of irradiation.

One type of PTC relies solely on light‐to‐heat conversion, with a distinguished feature that there is no significant difference in the catalytic results at the same temperature, whether under light or in the dark. For convenience, we term this as PTC‐T (thermal‐dominant photothermal chemistry). PTC‐T follows the L–H–C strategy, where the chemical reactions are driven by heat that is converted from solar energy and the reaction mechanism can be expounded by thermochemistry. The major distinction may be that the heat collection and chemical reaction processes can be told apart in conventional L–H–C processes, where the high temperature of the solar reactor is usually obtained by applying a concentrator with high a concentration ratio (CR) and a receiver; while the light‐to‐heat conversion of PTC mostly owes to the unique light absorption property of the materials, generating heat in situ when exposed to irradiation. PTC‐T provides a scheme to replace traditional energy sources with solar energy in thermochemistry.^[^ ^22^ ^]^

In another type of PTC, there are not only thermal effects (deriving from internal light‐to‐heat conversion or external auxiliary heat sources), but also some non‐thermal effects. In this case, the function of irradiation cannot be completely ascribed to the temperature increase, and the presence of light will change the results even though the temperature remains the same. Here, we term this as PTC‐S (synergistic photothermal chemistry). The mechanism of PTC‐S can be much more complicated due to the interaction of thermal and non‐thermal effects. Precisely because of this, PTC‐S has the potential to obtain better reaction results than thermochemical processes, making it possible to achieve higher reaction rate and higher energy efficiency under relatively mild conditions. It is also an approach for solar cascade utilization. However, the instance in which these two effects hold each other back cannot be ruled out. To achieve the beautiful vision, comprehending how the thermal and non‐thermal effects act in chemical processes is necessary.

### 2.1 Non‐Thermal Effect in PTC

#### 2.1.1 The Mechanisms

**Figure 3:**
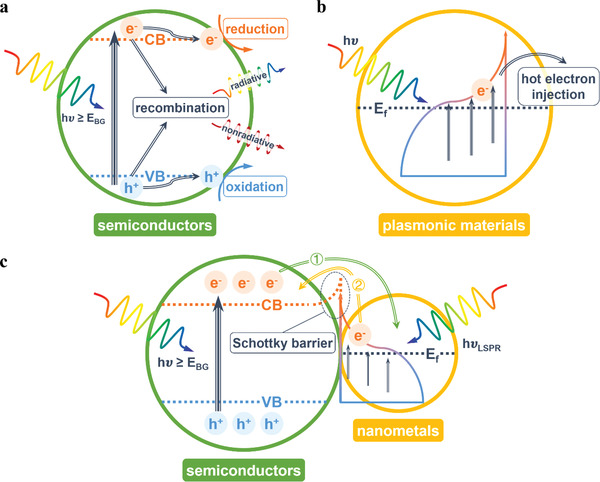
Photocatalytic mechanisms over a) semiconductors, b) plasmonic materials, and c) the composite of semiconductors and plasmonic nanometals.

To understand the mechanisms of non‐thermal effect, we have to expound the photocatalytic mechanism first. There are mainly two kinds of materials that can provide non‐thermal effect for reactions: semiconductors and plasmonic materials. Photocatalytic mechanism over semiconductors, as shown in **Figure** **3**a, is generally acknowledged. When light hits the surface of a semiconductor, electrons (e^−^) can be excited from the valence band (VB) to the conduction band (CB), leaving holes (h^+^) in the VB, if the energy of absorbed photons is greater than the bandgap energy (*E*_g_) of the semiconductor. They are usually called as photoinduced electron–hole pairs or photoinduced carriers. These energetic carriers can migrate to catalytic active sites, interact with reactant molecules or intermediate groups and drive the redox reactions. However, the photoinduced electron–hole pairs are likely to recombine and decay back to their ground state, releasing the excess energy in a radiative way or a nonradiative way.

Compared with semiconductors, plasmonic materials show different optical properties. When the frequency of the incident photons matches the intrinsic frequency of the surface electrons oscillating against the restoring force of positive nuclei, a resonant photoinduced collective oscillation of free electrons will occur, which is known as surface plasmon resonance (SPR) effect.^[^ ^23^ ^]^ SPR effect can enhance light absorption dramatically. When the size of plasmonic materials is small enough (nanoscale), light absorption can be further enhanced around the resonant frequency by localized surface plasmon resonance (LSPR) effect, with the light collection and the subsequent processes limited in a small space. The excellent light absorption can excite “hot carriers,” whose energies are larger than those of thermal excitations at ambient temperatures. On a femtosecond timescale, Landau damping will take place. Through electron–electron scattering, electron energy will go through a redistribution process and a high‐effective‐temperature Fermi–Dirac electron distribution will be formed, as shown in Figure 3b.^[^ ^24^ ^]^ These electrons and holes with relatively high energies can be injected to nearby molecules or adjacent catalysts, potentially driving relevant chemical processes.

In addition to the transfer and utilization of hot carriers, the non‐thermal effect over plasmonic nanomaterials can also act via near‐field enhancement.^[^ ^25^, ^26^ ^]^ When the plasmon resonance is within the illuminating spectral range, the absorption cross‐section can be larger than the physical cross‐section, and thus the optical or electromagnetic near‐field is enhanced, which not only promotes the hot carrier formation within the plasmonic nanomaterials, but also directly influences the electron states of the nearby reactant species or other catalyst components (promoting activation or improving carrier excitation). Generally, metallic materials, especially coinage metals (Au, Ag, and Cu), have relatively high carrier concentration and show strong plasmonic effect, which are recognized as typical plasmonic materials. Actually, LSPR effect can also be observed over semiconductors, such as some nonstoichiometric metal oxides or sulfides and some other modified semiconductors. Oxygen vacancy doped metal oxides (WO_3‐_ *_x_*, MoO_3‐_ *_x_*) and cation vacancy doped metal chalcogenides (Cu_2‐_ *_x_* S, Cu_2‐_ *_x_* Se) of size less than 20 nm generally display LSPR in the range of 700–1250 nm, while much larger nanoparticles of Au or Ag are required to make it falling into this spectral range. Through chemical doping or postsynthetic methods, the resonant window of semiconductors can be tuned across a wide optical spectral range from visible to far‐infrared, which is a unique advantage over metals.^[^ ^27^ ^]^

The non‐thermal effect, in a sense, can be deemed to be carrier‐driven effect, since the carrier behavior plays an important role in this mode. The photo‐induced carriers on semiconductors and the hot carriers on plasmonic materials are essentially similar. Both of them are induced by light and energetic for chemical processes, and it is imperative to increase their quantities and prolong their lifetime to achieve higher efficiency. Many efforts have been made, including metal loading, element doping, defect engineering, geometric and morphologic controlling, heterojunction construction, and so on.^[^ ^28^ ^]^ In brief, the first task is to extend the range of spectral response to increase the number of energetic carriers, followed by the second task that is to trap the electrons and holes at different locations for further utilization.

Considering the light absorption of semiconductors and plasmonic materials can fall into discrepant spectral ranges, the combination of semiconductors and plasmonic materials is a potential strategy for producing more energetic carriers. Moreover, this kind of composite, especially semiconductors loaded with nanometals, can form a unique electron transfer path. Generally, the Fermi levels of metals are lower than those of n‐type semiconductors. When they contact closely, charge will undergo redistribution to form a new equalized Fermi level, which will cause the CB to bend at the interface area and form a Schottky barrier, resisting electron transfer from the nanometals to the semiconductor. With light excitation, the Fermi level of the semiconductor will be lifted, resulting in a net electron transfer from the semiconductor to metal nanoparticles and improving the carrier separation efficiency, as shown in Figure 3c route ①.^[^ ^29^ ^]^ However, when the nanometals show LSPR effect under illumination, the electron transfer can be reversed. The hot electrons generated in the metal nanoparticles will be injected into the neighboring semiconductor across the Schottky barrier, as shown in Figure 3c route ②. This mode of carrier migration can prolong the lifetime of the hot carriers, which can even make the lifetimes of the injected hot electrons two orders of magnitude longer than those of electrons excited by UV irradiation within the semiconductor.^[^ ^30^, ^31^ ^]^

When the semiconductor and plasmonic metals are excited simultaneously, there is a competition between the Schottky barrier and LSPR hot electron injection. The offset of these two kinds of electrons with opposite transfer directions may result in a negative effect. Lin et al. applied mixed UV and green light irradiation with different ratios to degrade methylene blue on Au/TiO_2_.^[^ ^32^ ^]^ The UV light can excite TiO_2_, while the green light matches with the LSPR absorption peak of Au nanoparticles. On the one hand, the hot electrons induced by LSPR on the surface of the Au nanoparticles would favor the photocatalytic reaction. On the other hand, these hot electrons might surmount the Schottky barrier, accelerating the recombination of UV excited electron–hole pairs in TiO_2_ and possibly impairing the overall photocatalytic performance. Therefore, a balance between semiconductor excitation and LSPR effect should be sought depending on the specific situation, which can be regulated by altering the light intensity and spectral range.^[^ ^32^, ^33^ ^]^

#### 2.1.2 Gains from Non‐Thermal Effect

According to plenty of PTC researches, benefits from non‐thermal effect usually involve 1) reducing the energy barriers, so that the initial reaction temperatures can be reduced,^[^ ^34^, ^35^, ^36^, ^37^, ^38^, ^39^, ^40^, ^41^ ^]^ 2) promoting the activation of reactants and/or intermediate species, and thus accelerating the reaction rate,^[^ ^36^, ^42^, ^43^, ^44^, ^45^, ^46^, ^47^, ^48^ ^]^ 3) adjusting the reaction paths, and changing the product selectivity,^[^ ^42^, ^49^, ^50^, ^51^, ^52^, ^53^, ^54^ ^]^ and 4) desorbing harmful surface species or keeping the catalytic active sites (such as oxygen vacancy or low valence state) at a stable state to keep catalysts activated.^[^ ^39^, ^40^, ^43^, ^55^, ^56^, ^57^ ^]^

**Figure 4:**
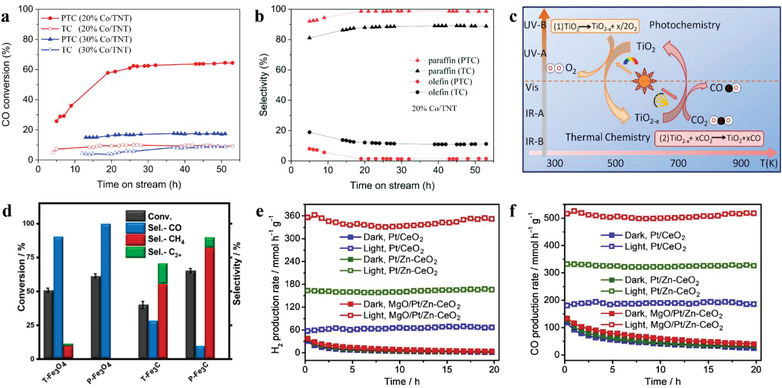
a) CO conversion against time and b) paraffin/olefin distribution of C_2_–C_4_ production under two catalytic conditions for 20% Co/TNT. Adapted with permission.^[^ ^42^ ^]^ Copyright 2018, The Royal Society of Chemistry. c) Schematic of photo‐thermochemical cycle for CO_2_ reduction. Reproduced with permission.^[^ ^58^ ^]^ Copyright 2015, Elsevier Ltd. d) Comparison of CO_2_ conversion results between photothermal catalysis (P) and thermal catalysis (T) on Fe‐based catalysts. Reproduced with permission.^[^ ^51^ ^]^ Copyright 2020, American Chemical Society. e) H_2_ production rates and f) CO production rates in the dark and under light irradiation at 600 °C for 20 h. Reproduced with permission.^[^ ^39^ ^]^ Copyright 2019, Elsevier B.V.

Attracted by the mild conditions of PC, researchers have considered introducing irradiation into TC processes, especially the reactions requiring high temperature. Fischer–Tropsch synthesis (FTS), converting syngas (CO and H_2_) into various hydrocarbons, is a typical TC process, which needs high temperature and high pressure. In order to develop a more energy‐saving and eco‐friendly FTS technology, Wang et al. combined thermo‐active components (Co) with photosensitive supports (TiO_2_ nanotube, TNT) and introduced UV illumination in FTS reaction.^[^ ^42^ ^]^ Under UV irradiation, photogenerated electrons are excited in TiO_2_ and then transfer to Co sites, which enhance the adsorption and activation of CO molecules at Co sites, showing a higher CO conversion rate (**Figure** **4**a). Moreover, the irradiation also promotes the hydrogenation of olefin and the hydrogenolysis of the long‐chain hydrocarbons, resulting in an increase in light paraffin selectivity, as shown in Figure 4b.

Two‐step thermochemical cycle for H_2_O splitting or CO_2_ reduction, consisting of a step of metallic oxide reduction and a step of H_2_O splitting or CO_2_ reduction, requires ultrahigh temperature, especially in the step of oxide reduction (usually over 1000 °C). Xu et al. have replaced the first high‐temperature thermochemical step with a photochemical process to lower the reaction temperature and improve solar energy efficiency.^[^ ^41^, ^58^ ^]^ The mechanism of photo‐thermochemical cycle over TiO_2_ is shown in Figure 4c. After loading Pd nanoparticles on the TiO_2_, an LSPR effect was introduced, which enhanced the light absorption in vis–NIR spectrum, provided more available charge carriers to induce more vacancies on TiO_2_, and achieved CO_2_ reduction at a temperature of less than 500 °C.^[^ ^41^ ^]^

Song et al. found that the CO_2_ conversion rate of PTC was higher than that of TC on the Fe‐based catalysts, while the product selectivity also varied due to the non‐thermal effect of light that changed the intermediate species and product formation/desorption capabilities (Figure 4d).^[^ ^51^ ^]^ When integrating photocatalysis and thermocatalysis for dry reforming of methane (DRM) on MgO/Pt/Zn–CeO_2_, Pan et al. found that the light illumination maintained the in situ generation of oxygen vacancy on CeO_2_ by photoinduced electrons, stabilizing the DRM process without deactivation. When the reaction was carried out in the dark, the production rates of H_2_ and CO showed severe deactivation, as shown in Figure 4e,f.^[^ ^39^ ^]^ It has also been found that the photoactivation can restrain CO disproportionation, the major side‐reaction of carbon deposition, thus decreasing the rate of carbon deposition during the process of DRM.^[^ ^57^ ^]^

In general, non‐thermal effect can remarkably promote some of those reactions that are thermodynamically nonspontaneous, making the reaction conditions relatively mild.

### 2.2 Thermal Effect in PTC

#### 2.2.1 The Mechanisms

**Figure 5:**
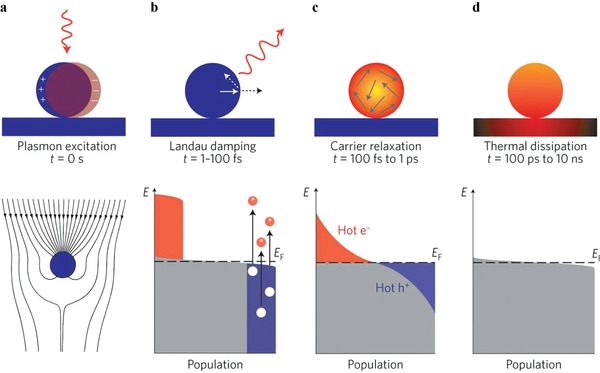
Photoexcitation and subsequent relaxation processes of metallic nanoparticles. a) The excitation of a localized surface plasmon redirects the flow of light (Poynting vector) toward and into the nanoparticle. b) In the first 1–100 fs following Landau damping, the athermal distribution of electron–hole pairs decays either through re‐emission of photons or through carrier multiplication caused by electron‐electron interactions. c) The hot carriers will redistribute their energy by electron–electron scattering processes on a timescale ranging from 100 fs to 1 ps. d) Heat is transferred to the surroundings of the metallic structure on a longer timescale ranging from 100 ps to 10 ns, via thermal conduction. Reproduced with permission.^[^ ^24^ ^]^ Copyright 2015, Nature Publishing Group.

There are commonly two sources of heat in PTC: one comes from the energy of irradiation, while the other comes from external heating. Although the photoinduced energetic carriers are competent to drive or promote chemical reactions, their short lifetimes limit their direct utilization efficiency in chemical reactions. Whether in semiconductors or plasmonic materials, radiative and/or nonradiative decay will take place shortly after the generation of energetic carriers. During the radiative decay, energy is emitted in the form of photons, which can be measured by photoluminescence spectroscopy. Along with the nonradiative relaxation of energetic carriers, electron–phonon scattering will take place after electron–electron scattering, and the energy of the electrons will dissipate into heat ultimately. In simple terms, the energy of carriers will be transferred to the phonons, which will intensify the lattice vibration and is manifest macroscopically as a temperature increase.^[^ ^29^ ^]^ This kind of local thermalization is more typical in plasmonic nanomaterials. On the one hand, the lifetimes of hot carriers are on the order of femtoseconds, as shown in **Figure** **5**, which are shorter than those of common semiconductors (on the scale of picoseconds to microseconds) and far shorter than the timescales (on the scale of milliseconds to seconds) required for reactions.^[^ ^24^, ^59^ ^]^ On the other hand, the main light absorption spectra of plasmonic nanomaterials usually fall into visible and near‐infrared region, which coincides with the main part of solar spectrum. Thus, the light‐to‐heat conversion over plasmonic nanomaterials can be remarkable, making them easier to integrate non‐thermal effect with thermal effect. Although the heat generation used to be considered as a side effect that had to be minimized, applying plasmonic nanoparticles as heat sources has attracted more and more interest.^[^ ^60^ ^]^

Besides, many organic materials can absorb light and convert it into heat through lattice vibration, especially when there are many *π* bonds in the structure. In graphene‐like allotropes, the large number of conjugated *π* bonds, that can provide various *π*–π* transitions, can promote light absorption at almost every wavelength of solar irradiation, and thus they generally present dark colors. After the *π*–π* transitions, the excited electrons will relax through electron–phonon coupling, leading to a temperature increase.^[^ ^21^ ^]^ Some specific nanoscale or mesoscale structures, such as nanowires, hollow spheres, core–shell structures, flower‐like structures, photonic crystals, and so on, can enhance light absorption at a broad spectral range, thereby enhancing the heat generation.^[^ ^44^, ^61^, ^62^, ^63^, ^64^, ^65^, ^66^, ^67^, ^68^ ^]^

#### 2.2.2 Light‐to‐Heat Conversion

**Figure 6:**
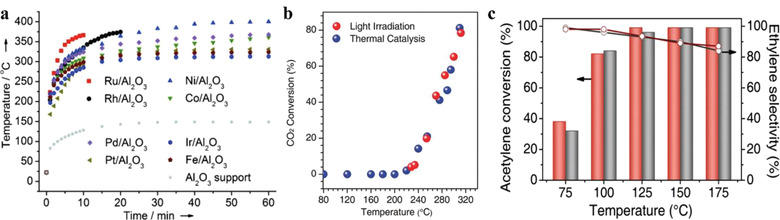
a) Temperature monitoring of the Group VIII catalysts under irradiation with a 300 W Xenon lamp. Reproduced with permission.^[^ ^69^ ^]^ Copyright 2014, Wiley‐VCH. b) Comparison of CO_2_ conversion for CoFe‐650 under photothermal heating (UV–vis irradiation) and direct thermal heating (no UV–vis irradiation). Reproduced with permission.^[^ ^71^ ^]^ Copyright 2017, Wiley‐VCH. c) Comparison of acetylene conversion and ethylene selectivity for Pd_1_/N–graphene under photothermal heating (UV–vis irradiation, red bar and red line) and direct thermal heating (no UV–vis irradiation, gray bar and gray line). Reproduced with permission.^[^ ^72^ ^]^ Copyright 2019, Wiley‐VCH.

With suitable compositions and structures, these materials can absorb light and convert it into heat efficiently. Under adequate light intensity, the generated heat suffices to replace the external heating required for thermocatalysis processes, which has been proved by several photothermal researches. Meng et al. prepared a series of Group VIII nanocatalysts for CO_2_ methanation.^[^ ^69^ ^]^ Under continuous irradiation with a 300 W Xenon lamp, all the Group VIII catalysts could maintain a reaction temperature of ≈300–400 °C (**Figure** **6**a) and present no obvious differences when heated by an oil‐bath without irradiation. Later, the same group also revealed that the photothermal reaction over Ru@FL‐LDHs was very similar to the traditional thermocatalysis.^[^ ^70^ ^]^ Preparing and applying a series of CoFe alloy catalysts for CO_2_ reduction, Chen et al. have found that the CO_2_ conversion at different reaction temperatures almost overlapped, no matter heated by irradiation or by external heating (Figure 6b).^[^ ^71^ ^]^ The estimated temperatures of CoFe‐650 under UV–vis irradiation have also been found to show a strong linear relationship with the Xe lamp intensity. Zhou et al. have synthesized atomically dispersed Pd single‐atom catalysts on nitrogen‐doped graphene (Pd_1_/N–graphene) for selective hydrogenation of C_2_H_2_ to obtain C_2_C_4_, which showed excellent property of light absorption at a wide spectral range.^[^ ^72^ ^]^ By varying the light intensity, the catalyst temperature could be regulated. However, the conversion of C_2_H_2_ and the selectivity to C_2_H_4_ with the two different heating methods (heated by the UV–vis light or other heat source without irradiation) showed no significant difference, as shown in Figure 6c.

#### 2.2.3 Gains from Thermal Effect

Even though light‐to‐heat conversion is an attractive alternative to other heating methods that consume fossil energy, the synergy of thermal and non‐thermal effects is more preferable, since they may complement and promote each other. Investigations reveal that thermal effect in a synergistic scenario can boost reactions via 1) facilitating the reaction kinetics,^[^ ^64^, ^73^, ^74^, ^75^, ^76^, ^77^ ^]^ 2) improving the mass transfer,^[^ ^75^, ^78^, ^79^ ^]^ 3) desorbing the products or harmful species,^[^ ^74^, ^80^, ^81^ ^]^ 4) forming beneficial thermal gradients under irradiation,^[^ ^82^, ^83^ ^]^ and 5) assisting carrier excitation.^[^ ^84^, ^85^, ^86^ ^]^

**Figure 7:**
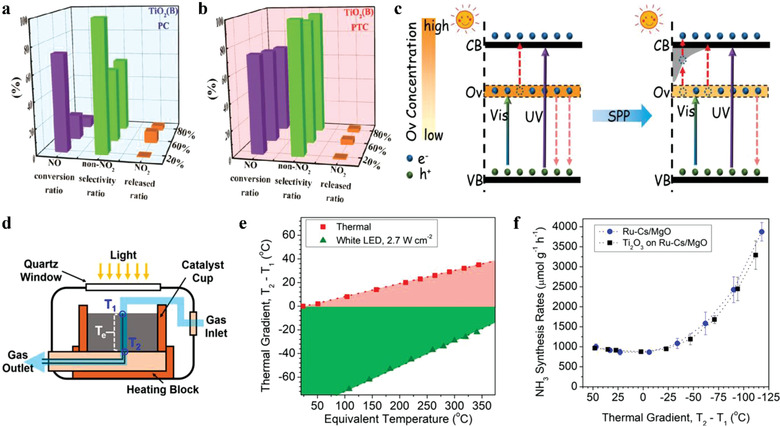
NO conversion, non‐NO_2_ selectivity, and NO_2_ release ratio of TiO_2_(B) microspheres in a) PC and b) PTC experiments. Reproduced with permission.^[^ ^80^ ^]^ Copyright 2020, Dalian Institute of Chemical Physics, the Chinese Academy of Sciences. Published by Elsevier B.V. c) Schematic illustration of the electron–hole separation mechanism for T‐0 h and T‐2 h samples during photocatalysis driven by UV and visible light irradiation. Reproduced under the terms of the Creative Commons CC BY license.^[^ ^85^ ^]^ Copyright 2020, The Authors. Published by Wiley‐VCH. d) Schematic representation of reaction chamber for in situ measurements of top‐ (T_1_) and bottom‐ (T_2_) temperatures of the catalyst bed. e) Measured thermal gradients under dark thermal (red squares) and heated white light illumination (green triangles). f) Measured NH_3_ synthesis rates on Ru‐Cs/MgO as a function of the thermal gradient for *T*_e_ = 325 °C under direct (blue circles) and indirect (black squares) illumination. Reproduced with permission.^[^ ^82^ ^]^ Copyright 2019, American Chemical Society.

According to the conventional view, temperature rise is detrimental to the separation of photoinduced carriers, and thus most of photocatalysis are usually conducted without IR irradiation at room temperature or even a lower temperature.^[^ ^87^ ^]^ Nevertheless, the temperature influence on photochemical process has aroused concern.^[^ ^46^, ^75^, ^87^, ^88^, ^89^ ^]^ Li et al. investigated photocatalytic water splitting over Au/N‐P25‐620/MgO (111) at elevated temperatures.^[^ ^89^ ^]^ The water‐splitting activity dramatically increased at high temperature and peaked at 270 °C, presenting extremely high quantum efficiencies, which coincided with the change trend of water dissociation constant. By coupling heat into photocatalysis, Peng et al. have found that the oxidation ability of lattice oxygen could be enhanced at the temperature of 60 °C, making the acetaldehyde degradation and CO_2_ generation more efficient.^[^ ^46^ ^]^ Many semiconductor oxides, used for photocatalytic removal of NO*_x_* species, tend to be deactivated at high relative humidity levels. Ma et al. found that the NO conversion ratio and non‐NO_2_ selectivity ratio on TiO_2_(B) dropped when the relative humidity increased from 20% to 80% under PC condition (with UV irradiation at room temperature), while these two ratios did not show a significant decrease under PTC condition (with UV irradiation at 60 °C), as shown in **Figure** **7**a,b.^[^ ^80^ ^]^

The thermal energy can also be obtained from the irradiation. Xu et al. combined graphene with great photothermal property with TiO_2_, proving that the elevation of surface temperature by graphene had a positive effect on the CO_2_ conversion since there was no decrease in the apparent activation energy.^[^ ^75^ ^]^ Etching to form vertically aligned nanowires on a Si wafer can dramatically enhance its light harvesting capability. In a study by Ozin's group, a vertically aligned silicon nanowire support was evenly coated by In_2_O_3‐_ *_x_* (OH)*_y_* nanoparticles, showing great performances in the light‐to‐heat conversion and the photoinduced charge carrier production, which can reduce CO_2_ to CO with irradiation as the only energy source.^[^ ^61^ ^]^

Some of the benefits from the thermal effect, such as kinetic promotion and mass transfer enhancement, are also common in thermochemistry, which can be provided by other heating methods. However, some benefits can only be obtained under irradiation. Li et al. measured the top temperatures (*T*_1_) and the bottom temperatures (*T*_2_) of the catalyst bed, and found that the irradiation above the catalyst could provide a thermal gradient opposite to that formed by external heating from the bottom, as shown in Figure 7d,e.^[^ ^82^ ^]^ The difference in temperature gradients is responsible for the enhancement of ammonia synthesis over ruthenium‐based catalyst. Even though the equivalent catalyst temperatures (*T*_e_) were set at the corresponding levels, ammonia synthesis rates under irradiation were higher than those heated in the dark, but it had nothing to do with the illumination wavelength. Later, a thin layer of Ti_2_O_3_, a black photothermal material that is inactive for ammonia synthesis, was placed on top of the catalyst to eliminate non‐thermal effect and create the same temperature gradient as that under direct irradiation. As shown in Figure 7f, the NH_3_ synthesis rate did not show significant difference whether or not exposed to irradiation directly, but had a strong dependence on the temperature gradient. This nonisothermal environment, acting as a thermodynamic pump, makes it possible to achieve conversion yield greater than the calculated equilibrium conversion by balancing the conflicting requirements of kinetics and thermodynamics. Yu et al. created shallow‐level defects above the deep‐level defects in TiO_2_ by a solution plasma processing technique.^[^ ^85^ ^]^ They found that the heat input helped to activate the migration of trapped electrons out of the deep‐level defects via these shallow levels (schematic illustration of the mechanism is shown in Figure 7c), which could improve both CO_2_ reduction and acetaldehyde degradation. In plasmonic semiconductor systems, Hattori et al. proved the operation temperature increase could improve hot electron harvesting, related to an enhancement of hot carrier generation caused by phonon coupling.^[^ ^86^ ^]^

Generally, elevated temperatures can overcome the kinetically sluggish to accelerate the reaction rate. Some reactions with obvious downhill characteristics (∆*G* ^0^ < 0), such as gas phase aerobic oxidation and some liquid‐phase organic degradations or transformations, can feasibly proceed at room temperature, which would be very sensitive to the thermal effect even though the high temperatures may sometimes be detrimental in thermodynamics.^[^ ^29^ ^]^ Thermal effect can also influence the carrier behavior and make an interaction with non‐thermal effect, presenting a synergistic result. Besides, compared to traditional heating, the thermal effect induced by irradiation can bring unexpected benefits, such as unique temperature gradients.

### 2.3 Possible Adverse Scenarios

In the foregoing discussion, we default that thermal and non‐thermal effects have positive effects on each other and promote chemical reactions together. Actually, they can handicap each other. It is well‐known that most reactions on semiconductors exhibit negative relationships between the reaction rate and temperature, because raising temperature may accelerate the recombination of carrier when there is no efficient carrier transfer path.^[^ ^23^, ^87^ ^]^ Westrich et al. proved that the decreasing reaction rates at an overhigh temperature were attributed to the nonradiative, multiphonon recombination of photogenerated charge carriers in calcined TiO_2_.^[^ ^90^ ^]^ As for plasmonic metals, Mahmoud found that the oxidation rate of DMAB over Ag nanotetrahedron arrays was lowered upon increasing the intensity of the excitation light, due to the thermalization of hot electrons leading to the desorption of reactant molecules.^[^ ^91^ ^]^ Sometimes the light‐to‐heat conversion results in the deactivation of catalysts, and sometimes the non‐thermal effect does a disservice in product selectivity. The photopromoted hydrogenation of olefin will decrease the selectivity of light olefins and increase the paraffin selectivity, which makes direct irradiation unfavorable when olefins are the target products.^[^ ^42^, ^92^ ^]^ Therefore, PTC catalysts should be carefully designed, and it is not wise to apply “photothermal” blindly.

## 3 Important Issues in PTC

### 3.1 Distinguishing and Quantifying Thermal and Non‐Thermal Effects

In a PTC process, especially that involves both thermal and non‐thermal effects, how to distinguish and quantify these two effects is of great importance but also of great difficulty. First of all, separating these two effects is necessary for understanding the precise mechanisms of reaction and energy conversion process thoroughly. The mechanism understanding can facilitate the rational design of PTC catalysts and efficient optimization of reaction conditions (such as searching for optimal irradiation intensity and spectrum, whether to employ auxiliary heating/cooling).

#### 3.1.1 Experimental Strategies

In order to distinguish the thermal and non‐thermal effects in PTC researches, it is common to set a series of comparative experiments, including photothermal condition (at a high temperature with irradiation), thermal condition (at the same temperature as photothermal condition but without irradiation) and photocatalytic condition (keeping at room temperature under irradiation). Baffou et al. have also summarized seven simple experimental procedures to distinguish photothermal from hot‐carrier effect in plasmonics, including 1) varying the illumination power, 2) varying the light beam diameter, 3) infrared and thermocouple measurements, 4) minding time scales, 5) calibrating with bubble formation, 6) comparing the effects of two polarizations, and 7) comparing the effects of several wavelengths.^[^ ^93^ ^]^

The reaction results of PTC are often found to be much higher than the simple sum of photocatalysis and thermocatalysis.^[^ ^45^, ^88^ ^]^ This comparison can only elucidate the thermal effect and the non‐thermal effect qualitatively, far from decoupling and quantifying them, because the interaction of these two effects does not simply present as an algebra sum. The differences or ratio of the reaction rates at the same temperature with or without irradiation can reflect the photopromotion at this temperature to some extent.^[^ ^34^, ^39^ ^]^ The differences in turnover frequency (TOF) or apparent activation energies under light or dark conditions can also be applied to indicate the photopromotion.^[^ ^35^, ^37^, ^94^ ^]^ To further demonstrate the light efficiency, it can be assumed that the difference in reaction rates results from the irradiation and the light efficiency can be calculated by Equation (1)^[^ ^34^ ^]^

(1) $$\begin{array}{ccc} & & \eta_{\mathrm{energy}\;\mathrm{efficiency}}\;\left(\%\right)\\ & & \;=\frac{\mathrm{Difference}\;\mathrm{in}\;\mathrm{reaction}\;\mathrm{rate}\;\left(\mathrm{Light}-\mathrm{Dark}\right)\times \Delta H_{\mathrm{reaction}}}{\mathrm{Intensity}\times \mathrm{Catalyst}\;\mathrm{surface}\;\mathrm{area}}\\ & & \;\times \;100\% \end{array}$$

or by Equation (2)

(2) $$\begin{array}{ccc} & & \eta_{\mathrm{quantum}\;\mathrm{efficiency}}\;\left(\%\right)\\ & & \;=\frac{\mathrm{Difference}\;\mathrm{in}\;\mathrm{reaction}\;\mathrm{rate}\;\left(\mathrm{Light}-\mathrm{Dark}\right)\times \Delta n_{\mathrm{electron}}}{\mathrm{Photon}\;\mathrm{flux}\times \mathrm{Catalyst}\;\mathrm{surface}\;\mathrm{area}}\\ & & \;\times \;100\% \end{array}$$

where the ∆*H*_reaction_ is the enthalpy of reaction, and ∆*n*_electron_ is the electron transfer number of the reaction.

As for the thermal effect, measuring the temperature rise under irradiation is a common method to illustrate the light‐to‐heat conversion capability of PTC catalysts. However, it is quite difficult to figure out a definite efficiency, since the reaction heat and phase transformation latent heat of some reactants are also mingled in the PTC processes. Besides, testing the thermal activity in the dark can reflect the temperature influence to some extent. Some of the chemical reactions can be simply driven by heat, while some of them need irradiation as the major driving force.

#### 3.1.2 Temperature‐Related Challenges

There is a crucial problem in control experiments: the accuracy of temperature measurement and reproduction. Some researchers believe that heat generation in nanomaterials, especially in those with plasmonic effect, will show a localized nature, causing the impossibility to measure the accurate temperature in nanoscale. Nanoscale makes the macroscopic definition of temperature inapplicable, and also makes the measure results more sensitive to the invasive interactions between the materials and the temperature probe.^[^ ^10^ ^]^ Cai et al. proposed a method for estimating the local temperature by deducing backward from the composition of different gases when the reactions reached the equilibrium state.^[^ ^95^ ^]^ This estimation method is applicative for the PTC‐T processes, while in the PTC‐S processes, non‐thermal effect makes the relationship between equilibrium state and temperature more intricate. The estimated temperature is more like an equivalent temperature rather than the temperature in a conventional sense. Actually, the PTC processes usually take place under continuous illumination, and the temperature increase will be spread via heat transfer.^[^ ^93^ ^]^ The temperature difference on the surface of PTC catalysts is not so much as envision, but far from ignorable. In general, the temperature gradient in a gas–solid heterogeneous reaction system will be more significant than that in a liquid‐phase reaction system, due to the poorer heat transfer and higher temperature on the surface of catalysts.^[^ ^82^ ^]^

The choice of temperature measurement methods is also of importance. The most common method for temperature measurement, thermocouple, can reflect only the temperature at a single point and exhibit a time delay due to thermal equilibrium, which will cause a deviation in control experiments; while the temperature measurement method using infrared camera can measure surface temperature distributions, but sometimes affected by the transmittance of the reactor windows and the emissivity of heterogeneous materials.^[^ ^96^ ^]^ With advances in measurement technology, such as scanning thermal microscope, surface‐enhanced Raman spectroscopy (SERS), tip‐enhanced Raman spectroscopy, scanning transmission electron microscopy, and so on, temperature measurement with higher spatial and temporal resolution can be achieved.^[^ ^97^, ^98^, ^99^, ^100^ ^]^ However, the temperature profiles under irradiation are difficult to reproduce by other heating methods.

When calculating the light efficiency according to Equation (2), Liu and co‐workers found an unreasonable quantum efficiency of around 800% over Ru/TiO_2_ for CO_2_ methanation, which indicated that a hot electron created by a photon could be used for reaction more than once.^[^ ^101^ ^]^ It was because the temperature on top of catalyst bed (*T*_1_) would increase under irradiation, while the heating was controlled by the temperature of chamber (*T*_c_). Thus, the thermal gradient formed by light‐to‐heat conversion was not taken into account when taking *T*_c_ to represent the reaction temperature. To figure out the temperature profile of the catalyst bed, they applied multiple inserted thermocouples to sketch the temperature gradient of the catalyst bed, assuming that non‐thermal reaction was confined in a thin layer at the top of catalyst bed and an equivalent temperature (*T*_e_) was calculated out by Equation (3) to represent the thermal reaction temperature

(3) $$e^{\frac{-E_{a}}{RT_{e}}}=\frac{1}{T_{2}-T_{1}}\;\int_{T_{1}}^{T_{2}}e^{\frac{-E_{a}}{RT}}dT$$

where *E*_a_ is the apparent activation energy, *R* is the molar gas constant, *T*_1_ and *T*_2_ are the top temperature and the bottom temperature of the catalyst bed, respectively.

**Figure 8:**
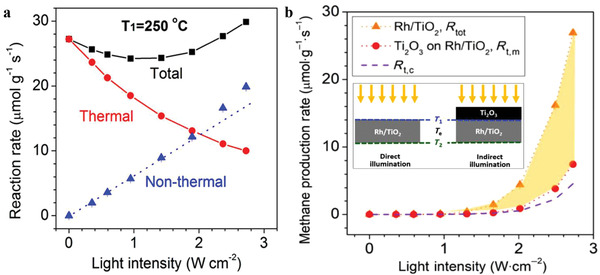
a) Total (black squares), thermal (red circles), and non‐thermal (blue triangles) reaction rates at T_1_ = 250 °C as a function of *I*_uv_ (the non‐thermal reaction rate is the difference between the total and effective thermal reaction rates). Reproduced with permission.^[^ ^101^ ^]^ Copyright 2018, American Chemical Society. b) Comparation of measured total CH_4_ production rate (*R*_tot_, orange triangles) as a function of UV light intensity, calculated (*R*_t,c_, purple dashes) thermal CH_4_ production rates based on corresponding *T*_e_ and measured (*R*_t,m_, red circles) thermal CH_4_ production rate under indirect illumination with identical thermal profiles, when the top surface temperature is kept at 250 °C (the inserted figure illustrates the two illumination conditions). Adapted with permission.^[^ ^102^ ^]^ Copyright 2019, Tsinghua University Press and Springer Nature.

In this way, the thermal reaction rate (*R*_t_) could be calculated and the non‐thermal reaction rate (*R*_nt_) could be obtained by subtracting *R*_t_ from the total measured reaction rate as shown in **Figure** **8**a, with a more reasonable quantum efficiency around 5.4–5.6% at 300 °C. In one of their subsequent researches, a novel indirect illumination technique was proposed.^[^ ^102^ ^]^ By covering a layer of inactive light‐absorbing materials on the top of catalyst, an indirect illumination condition was created, which could form the same temperature gradient as that under direct illumination and eliminate non‐thermal effect in the meantime. By this novel method, the thermal CH_4_ production rates could be captured via experiment measurement and the non‐thermal contribution could be extracted, as shown in Figure 8b. Moreover, the apparent quantum efficiency, calculated from the non‐thermal reaction rate, was found to show a striking dependence on the top surface temperature. This indirect illumination method was also used in the ammonia synthesis, which proved that the unique temperature gradient formed by irradiation was beneficial to the NH_3_ production (having been mentioned in Section 2.2.3).^[^ ^82^ ^]^

#### 3.1.3 In Plasmonic Materials

**Figure 9:**
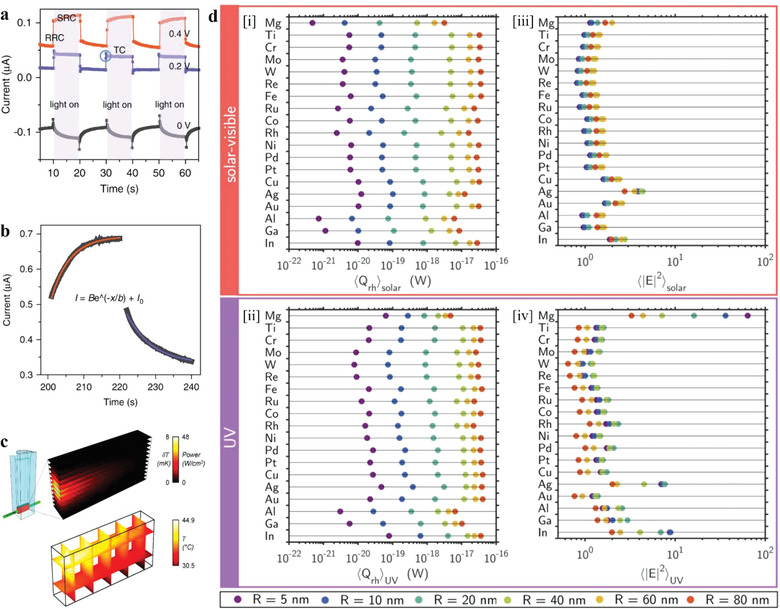
a) The photocurrent of the Au nanoelectrode array at different applied potentials, which can be divided in two parts according to the response time: the RRC (0.05 s) and the SRC (10 s). b) The comparation of the fitted SRC (red line: under illumination, blue line: in the dark) and the experimental result (black dot) at 0.6 V, 200–240 s. Reproduced under the terms of the CC BY 4.0 license.^[^ ^103^ ^]^ Copyright 2019, The Author. Published by Springer Nature. c) The 3D spatial distribution of the absorbed optical power per unit cell and the local temperature increase (the upper); steady‐state temperature profile inside the nanoparticle solution due to collective heating effects, after 30 min of laser irradiation (the lower). Reproduced with permission.^[^ ^96^ ^]^ Copyright 2018, American Chemical Society. d) Heat power delivered, averaged over [i] 〈*Q*_rh_〉_solar_ and [ii] 〈*Q*_rh_〉_UV_ spectral ranges; near‐field enhancement averaged over the NPs surface and over [iii] 〈*|E|^2^* 〉_solar_ and [iv] 〈*|E|^2^* 〉_UV_ spectral ranges (different colors represent magnitudes calculated for spherical NPs with different radii *R*). Reproduced with permission.^[^ ^26^ ^]^ Copyright 2020, American Chemical Society.

Owing to both excellent hot carrier generation and photothermal effect, there are quite a few researches focused on distinguishing and quantifying these two effects in plasmonic materials.^[^ ^16^, ^26^, ^37^, ^93^, ^96^, ^101^, ^102^, ^103^ ^]^ Zhan et al. quantitatively disentangled the influence of increased temperature from energetic carrier effects via a photoelectrochemistry method.^[^ ^103^ ^]^ The plasmonic photocurrent could be divided in two parts according to the response time: the rapid response current (RRC, 0.05 s) and the slow response current (SRC, 10 s), as shown in **Figure** **9**a. According to the relationship between current and temperature at a certain voltage (fitted by Equation (4)) and the temperature variation against the irradiation time (calculated by Equation (5) based on the linear nonequilibrium thermodynamics), the current–time equation can be derived. The calculated current–time curves are shown in Figure 9b, which matches well with the experimental SRC. Besides, the RRC showed a superlinear dependence on the incident light intensity, with a wavelength‐dependence corresponded with the UV–vis extinction spectrum (LSPR part); while the SRC shows a linear dependence and decreases with the increasing of wavelength. All the evidences prove that the RRC and SRC can reflect the carrier effect and photothermal effect, respectively

(4) $$I\;=A_{0}\;T+D$$

(5) $$T\;=\;Ae^{-\frac{ak}{Cl}t}+\frac{Pl}{ak}+T_{0}$$

where *I* is the current, *A*_0_ and *D* are constants that can be obtained from the measured current as the function of temperature, *A* is a constant determined from boundary conditions, *a* is the size of the electrode, *k* is the thermal conductivity, *C* is the heat capacity of the system, *l* is the thickness of the thermal diffusion layer in which the temperature changes linearly, *t* is the time, *P* is the energy input by the incident light, and *T*_0_ is the external temperature considered constant.

Some more refined characterization techniques are also used to solve this task, such as scanning electrochemical microscopy, SERS, and in situ thermal coupled photoconductivity.^[^ ^84^, ^104^, ^105^ ^]^ In addition, numerical modeling is a helpful technique to obtain some information, such as 3D temperature distribution and electric field enhancement, which are impossible or hard to obtain via experimental methods. Kamarudheen et al. chose a temperature‐sensitive synthesis of Au@Ag core@shell nanoparticles as the probe reaction and used numerical modeling to simulate light propagation and heat transfer, obtaining the 3D temperature distributions of the reaction system under experimental conditions, as shown in Figure 9c.^[^ ^96^ ^]^ The shell growth rates were observed under light irradiation and in the dark, respectively, compared with the numerical results as well, to quantify the contributions of photothermal and hot charge carrier effects. The LSPR‐induced enhancement of electric field intensity can be simulated via numerical methods, such as finite‐difference time‐domain, discrete dipole approximation, finite element method (FEM) of COMSOL, and so on. ^[^ ^26^, ^36^, ^41^, ^48^, ^79^, ^94^, ^106^ ^]^

Gutierrez et al. modeled the interaction of light with 19 metals NPs by means of FEM simulations using COMSOL Multiphysics software, and then defined and calculated two figures of merit, *Q*_rh_ and *|E|* ^2^, on nanospheres with radii (*R*) ranging from 5 to 80 nm under the irradiation of solar‐visible or UV light respectively, as shown in Figure 9d.^[^ ^26^ ^]^ The figure of *Q*_rh_ is to evaluate the ability of photothermal heat generation, while the indicator of *|E|* ^2^ is to quantify the near‐field enhancement. By comparing 〈*Q*_rh_〉_solar_, 〈*Q*_rh_〉_UV_, 〈*|E|* ^2^〉_solar_, and 〈*|E|* ^2^〉_UV_ of the 19 selected metals with different radii, the photothermal and non‐thermal properties of these 19 metals were revealed clearly.

To make a unified quantifying of PTC performance incorporating both photochemical and thermal effect, Ozin and co‐workers have defined a temperature‐dependent dimensionless photothermal figure of merit, PTF(*T*)^[^ ^10^ ^]^

(6) $$\mathrm{PTF}\;\left(T\right)=\eta_{\mathrm{CT}}\;\left(T^{*}\right)\alpha \frac{\kappa_{\mathrm{int}}\left(T^{*}\right)T^{*}}{\kappa_{\mathrm{cat}}\left(T^{*}\right)T}$$

where *α* is the solar absorptance, *η*_CT_(T*) is the photochemical charge transfer efficiency at temperature T*, *κ*_int_ is the thermal conductivity of the interface between the nanoheater and the catalyst, *κ*_cat_ is the thermal conductivity of the catalyst substrate, T* is the local, nonequilibrium temperature of the catalyst, and *T* is the measured temperature of the catalyst bed.

This figure of merit can be calculated as a comprehensive assessment of the PTC catalyst. However, some parameters in the equation are difficult to obtain, making it little practicable in application. Directly calculating the total efficiency of energy conversion may be more feasible, just needing to make a reasonable consideration about what should be included in the numerator and denominator of the energy efficiency.

### 3.2 From Academic Studies to Engineering Applications

**Figure 10:**
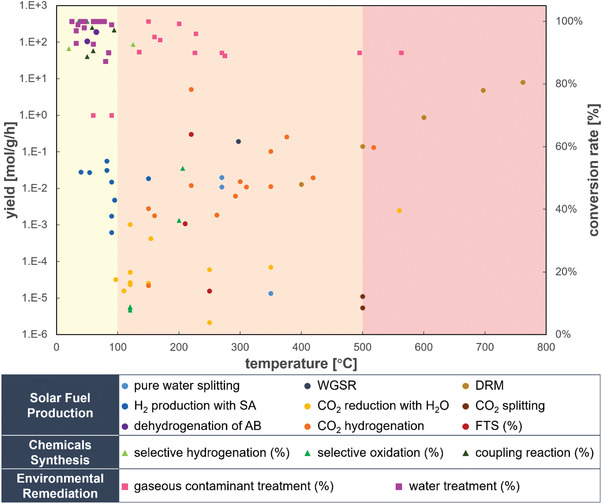
The relationship between the temperatures and reaction results in different PTC processes (the legends without “(%)” correspond with the primary *y*‐axis, while the legends with “(%)” correspond with the secondary *y*‐axis).

We have summarized a number of representative PTC works involving different fields, as shown in **Table** **1**.^[^ ^33^, ^36^, ^38^, ^39^, ^40^, ^41^, ^42^, ^43^, ^44^, ^45^, ^46^, ^47^, ^49^, ^50^, ^51^, ^52^, ^53^, ^54^, ^55^, ^56^, ^57^, ^61^, ^62^, ^63^, ^64^, ^65^, ^66^, ^67^, ^68^, ^70^, ^71^, ^72^, ^73^, ^75^, ^76^, ^77^, ^78^, ^79^, ^80^, ^81^, ^83^, ^85^, ^87^, ^88^, ^89^, ^94^, ^111^, ^112^, ^113^, ^115^, ^117^, ^118^, ^120^, ^121^, ^123^, ^124^, ^125^, ^126^, ^127^, ^128^, ^129^, ^130^, ^131^, ^132^, ^133^, ^134^, ^135^, ^136^, ^137^, ^138^, ^139^, ^140^, ^141^, ^142^, ^143^, ^144^, ^145^, ^146^, ^147^, ^148^, ^149^, ^150^, ^151^, ^152^, ^153^, ^154^, ^155^, ^156^, ^157^, ^158^, ^159^, ^160^, ^161^, ^162^, ^163^, ^164^, ^165^, ^166^, ^167^, ^168^ ^]^ The PTC works are categorized into three major fields, solar fuel production, chemical synthesis, and environmental remediation, and the catalysts and energy sources used in the studies, along with the reaction temperatures and reaction results are collected. Besides, the datasets of the temperatures and the reaction results have been extracted and plotted for better visibility, as shown in **Figure** **10**. There are mainly liquid‐phase reactions in the light‐yellow region with temperature under 100 °C. The temperatures of most of PTC reactions are less than 500 °C, which corresponds with the mid‐and‐low temperature solar energy utilization.^[^ ^107^ ^]^ Except for degradation of gaseous or water pollutants, dehydrogenation of ammonia borane (AB) and high‐temperature DRM, quite a few of PTC researches are still at a laboratorial stage with small scales and low yields.

**Table 1:** Representative PTC works in different fields

Application	Catalyst	Energy source	Temperature^a)^ [°C]	Reaction result^b)^ (conversion rate/production rate/selectivity/efficiency)	Ref.
Solar fuel production					
Pure water splitting	Au/N‐P25/MgO (111)	Tungsten lamp (Vis, 0.45 kW m^−2^) and external heating	270	H_2_: 11 mmol g^−1^ h^−1^	^[^ ^88^ ^]^
		Four halogen lamps	270	H_2_: 20 mmol g^−1^ h^−1^	
	Ni/Cu–TiO_2_	Xe lamp (<760 nm, 6 kW m^−2^) and external heating	350	H_2_: 13.50 µmol g^−1^ h^−1^	^[^ ^123^ ^]^
Hydrogen production from water with sacrificial agents (SA)	Ag/MoS_2_/TiO_2‐_ *_x_*	Xe lamp (>420 nm)	/	H_2_: 1.98 mmol g^−1^ h^−1^	^[^ ^124^ ^]^
	Au/TiO_2_	Xe lamp (15 kW m^−2^)	82	H_2_: 56.25 mmol g^−1^ h^−1^ integrated with PV (total SE: 4.2%)	^[^ ^120^ ^]^
	Cu/Al_2_O_3_/ZnO	Solar irradiation (1 kW m^−2^ with parabolic reflector)	180	CO & H_2_; SE: 67.49% integrated with photochemical energy storage (total SE: 75.38%)	^[^ ^117^ ^]^
			/	CO & H_2_; SE: 45.17% integrated with photochemical energy storage and PV (total SE: 66.95%)	^[^ ^118^ ^]^
	Cu/TiO_2_	Xe lamp and external heating	90	H_2_: ≈ 15 mmol g^−1^ h^−1^	^[^ ^125^ ^]^
	Cu_2‐_ *_x_* S/CdS/Bi_2_S_3_	Xe lamp (>420 nm)	/	H_2_: 8.012 mmol g^−1^ h^−1^	^[^ ^126^ ^]^
	NiS@g‐C_3_N_4_	Xe lamp (>420 nm)	82.2	H_2_: 31.3 mmol g^−1^ h^−1^	^[^ ^127^ ^]^
	P25	Xe lamp (15 kW m^−2^)	90	H_2_: 1.736 mmol g^−1^ h^−1^ SE: 0.0005%	^[^ ^128^ ^]^
		Solar irradiation (Fresnel lens, 36 suns)	≈95	H_2_: 4.716 mmol g^−1^ h^−1^ SE: 0.022%	
	Pt/TiO_2_	LED (380–450 nm) and external heating	90	H_2_: ≈ 0.625 mmol g^−1^ h^−1^	^[^ ^88^ ^]^
	Pt/TiO_2_	Xe lamp (320–800 nm) and external heating	40	H_2_: 28.05 mmol g^−1^ h^−1^ QE: 203%	^[^ ^87^ ^]^
	Pt/TiO_2_	Xe lamp	54	H_2_: 27.07 mmol g^−1^ h^−1^ SE: 0.36%	^[^ ^129^ ^]^
	Pt@STO	Xe lamp (5.3 kW m^−2^)	150	95.5%/15 min CO: 11.44 mmol g^−1^ h^−1^; H_2_: 18.616 mmol g^−1^ h^−1^ syngas: 94.4%	^[^ ^130^ ^]^
Water–gas shift reaction (WGSR)	CuO*_x_*/ZnO/Al_2_O_3_	Simulated sunlight (1 kW m^−2^)	297	H_2_: 192.33 mmol g^−1^ h^−1^	^[^ ^113^ ^]^
		Solar irradiation (0.16–0.42 kW m^−2^, 4.2 m^2^)	270–410	H_2_: 580–1240 L h^−1^ SE: 2.86%	
Dehydrogenation of ammonia borane (AB)	Ag/W_18_O_49_	Xe lamp (>750 nm, 54 W m^−2^)	55	10.8 µmol h^−1^	^[^ ^131^ ^]^
		Solar irradiation (5.50 kW m^−2^)	–	2.76 µmol h^−1^	
	RGO/Na_2_Ti_3_O_7_	Xe lamp (2.2 kW m^−2^)	Δ*T* = ≈40	H_2_: 189.7 mol g^−1^ h^−1^	^[^ ^132^ ^]^
	Ti_2_O_3_	Xe lamp (19 kW m^−2^)	≈195	H_2_: AB = 2.0/30 min	^[^ ^133^ ^]^
		Xe lamp (1 kW m^−2^) and waste heat of 70 °C and CuCl_2_ promoter	93	H_2_: AB = 2.0/30 min	
	TiN–Pt	Simulated sunlight (AM 1.5G, 10 kW m^−2^)	≈50	H_2_: 106.4 mol g_Pt_^−1^ h^−1^	^[^ ^94^ ^]^
CO_2_ reduction with H_2_O	3DOM‐LaSrCoFeO_6‐_ *_x_*	Xe lamp (>420 nm) and external heating	350	CH_4_: 69.735 µmol g^−1^ h^−1^ SE: 1.933%	^[^ ^73^ ^]^
	AuCu/g‐C_3_N_4_	Xe lamp (>420 nm) and external heating	120	CH_3_OH: 0.14 mmol g^−1^ h^−1^; CH_3_CH_2_OH: 0.89 mmol g^−1^ h^−1^, 93.1%	^[^ ^50^ ^]^
	Bi_2_S_3_/UiO‐66	Xe lamp (6.5 kW m^−2^)	150	CO: 25.60 µmol g^−1^ h^−1^, 99.0%	^[^ ^134^ ^]^
	Bi_4_TaO_8_Cl/W_18_O_49_	Xe lamp (<780 nm, 1.80 kW m^−2^) and external heating	120	CO: 23.42 µmol g^−1^ h^−1^	^[^ ^135^ ^]^
	Cu^0^/Cu_2_O	Xe lamp (4 kW m^−2^) and external heating	110	CO: 13.2 µmol g^−1^ h^−1^; CH_3_OH: 2.6 µmol g^−1^ h^−1^	^[^ ^136^ ^]^
	Cu/TiO_2_‐C	Xe lamp and external heating	250	CH_4_: 60 µmol g^−1^ h^−1^	^[^ ^55^ ^]^
	Fe_2_O_3_/Fe_3_O_4_	Solar irradiation (Fresnel lens, CR = 600)	560	CH_4_: 1470.7 µmol g^−1^ h^−1^; C_2_H_4_: 736.2 µmol g^−1^ h^−1^; C_2_H_6_: 277.2 µmol g^−1^ h^−1^ SE: 0.05%	^[^ ^137^ ^]^
	H‐Ov‐TiO_2_(AB)	Xe lamp (1 kW m^−2^) and external heating	120	CO: 38.99 µmol g^−1^ h^−1^; CH_4_: 11.93 µmol g^−1^ h^−1^	^[^ ^85^ ^]^
	m‐WO_3‐_ *_x_*	Xe lamp (>420 nm) and external heating	250	CH_4_: 2.148 µmol g^−1^ h^−1^ SE: 0.82%	^[^ ^47^ ^]^
	Pd/WN‐WO_3_	Xe lamp (AM 1.5G, 4 kW m^−2^)	154	H_2_: 368.5 µmol g^−1^ h^−1^; CO: 15.2 µmol g^−1^ h^−1^; CH_4_: 40.6 µmol g^−1^ h^−1^	^[^ ^138^ ^]^
	TiO_2‐_ *_x_*/CoO*_x_*	UV lamp (0.2 kW m^−2^) and external heating	120	CO: 16.403 µmol g^−1^ h^−1^; CH_4_: 10.051 µmol g^−1^ h^−1^	^[^ ^139^ ^]^
	TiO_2_‐G	Xe lamp (4.38 kW m^−2^)	96.5	CO: 5.2 µmol g^−1^ h^−1^; CH_4_: 26.7 µmol g^−1^ h^−1^	^[^ ^75^ ^]^
	TiO_2_ PC	Xe lamp	Δ*T* = ≈2	CH_4_: 35.0 µmol h^−1^ m^−2^	^[^ ^68^ ^]^
CO_2_ hydrogenation	Co/Al_2_O_3_	Xe lamp (13 kW m^−2^)	292	CO: 0.1392 mmol g^−1^ h^−1^, 2.3%; CH_4_: 6.036 mmol g^−1^ h^−1^, 97.7%	^[^ ^43^ ^]^
	Co@CoN&C	Xe lamp	518	41.3%/30 min CO: 132 mmol g^−1^ h^−1^, 91.1%	^[^ ^54^ ^]^
	CoFe–Al_2_O_3_	Xe lamp	310	82.2%/2 h CO: 2.97%; CH_4_: 60.61%; C_2+_: 36.42%	^[^ ^71^ ^]^
	Cu‐HAP	Xe lamp (40 kW m^−2^)	≈220	CO: 12 mmol g^−1^ h^−1^, >99%	^[^ ^38^ ^]^
	Fe_3_O_4_	Xe lamp (20.5 kW m^−2^)	350	CO: 11.3 mmol g^−1^ h^−1^, >99%	^[^ ^51^ ^]^
	Fe_3_C		310	CH*_x_*: 10.9 mmol g^−1^ h^−1^, 97.5%	
	FeO–CeO_2_	Xe lamp (22 kW m^−2^)	419	44.33% CO: 19.61 mmol g^−1^ h^−1^, 99.87%	^[^ ^140^ ^]^
	Ga–Cu/CeO_2_	Xe lamp (19.52 kW m^−2^)	280	CO: 111.2 mmol g^−1^ h^−1^, 100% SE: 0.83%	^[^ ^141^ ^]^
	In_2_O_3‐_ *_x_*	Xe lamp	≈350	CO: 103.21 mmol g^−1^ h^−1^	^[^ ^142^ ^]^
	In_2_O_3‐_ *_x_*	Xe lamp (≈20 kW m^−2^)	262	CO: 1.875 mmol h^−1^ m^−2^	^[^ ^143^ ^]^
	In_2_O_3‐_ *_x_* (OH)*_y_*	LED (380 nm, 43.4 kW m^−2^)	300	CO: 15.4 mmol g^−1^ h^−1^	^[^ ^115^ ^]^
	In_2_O_3‐_ *_x_* (OH)*_y_*/SiNW	Xe lamp (20 kW m^−2^)	150	CO: 22.0 µmol g^−1^ h^−1^	^[^ ^61^ ^]^
	Ni/BaTiO_3_	Xe lamp (2.93 kW m^−2^)	376	94.4%/10 min CH_4_: 257.0 mmol g^−1^ h^−1^, ≈100%	^[^ ^144^ ^]^
	Pd@Nb_2_O_5_	Xe lamp (25 kW m^−2^)	160	CO: 1.8 mmol g^−1^ h^−1^	^[^ ^145^ ^]^
	Ru/Al_2_O_3_	Simulated sunlight (6.2 kW m^−2^) and external heating	220	CH_4_: 5.09 mol g^−1^ h^−1^	^[^ ^83^ ^]^
	Ru@FL‐LDH	Xe lamp (10 kW m^−2^)	350	96.3% CH_4_: 99.3%	^[^ ^70^ ^]^
	Ru/i‐Si‐o	Xe lamp (24.7 kW m^−2^)	∼150	CH_4_: 2.8 mmol g^−1^ h^−1^	^[^ ^44^ ^]^
Dry reforming of methane (DRM)	MgO/Pt/Zn–CeO_2_	Simulated sunlight (30 kW m^−2^) and external heating	600	CO: 516 mmol g^−1^ h^−1^; H_2_: 356 mmol g^−1^ h^−1^	^[^ ^39^ ^]^
	NiCo/Co–Al_2_O_3_	Xe lamp	762	CO: 4231.8 mmol g^−1^ h^−1^; H_2_: 3807.6 mmol g^−1^ h^−1^ SE: 29.7%	^[^ ^146^ ^]^
	Ni–La_2_O_3_/SiO_2_	Xe lamp (8068.6 mW)	697	CO: 2574.0 mmol g^−1^ h^−1^; H_2_: 2286.6 mmol g^−1^ h^−1^ SE: 20.3%	^[^ ^57^ ^]^
	Pt–Au/SiO_2_	Xe lamp (300–800 nm, 6 kW m^−2^) and external heating	400	CO: ≈7.2 mmol g^−1^ h^−1^; H_2_: ≈5.7 mmol g^−1^ h^−1^; syngas: ≈100%	^[^ ^36^ ^]^
	Pt/TaN	Xe lamp (420–780 nm, 4.20 kW m^−2^) and external heating	500	CO: ≈75 mmol g^−1^ h^−1^; H_2_: ≈66 mmol g^−1^ h^−1^; syngas: ≈100%	^[^ ^147^ ^]^
CO_2_ splitting	Cu–TiO_2_	Hg lamp and external heating	500	CO: 5.40 µmol g^−1^ h^−1^	^[^ ^148^ ^]^
	PNT	Hg lamp and external heating	500	CO: 11.05 µmol g^−1^ h^−1^	^[^ ^41^ ^]^
Fischer–Tropsch synthesis (FTS)	Co/TiO_2_	Hg lamp and external heating	220	63.9% CO_2_: 3.1%; CH*_x_*: 96.9% (CH_4_: 35.0%; C_2_–C_4_: 36.3%; C_5+_: 28.7%)	^[^ ^42^ ^]^
	CoAl‐LDH	Xe lamp (200–800 nm)	210	35.4% CO_2_: 17.3%; CH*_x_*: 82.7% (CH_4_: 34.6%; C_2_–C_4_: 22.7%; C_5+_: 42.7%)	^[^ ^149^ ^]^
	CoMn*_x_*/MnO_2‐_ *_x_*	Xe lamp (34–39 kW m^−2^)	250	13.9%/30 min CO_2_: 22.6%; CH_4_: 28.4%; C_2_–C_4_ (olefins): 27.0%; C_2_–C_4_ (paraffins): 8.4%; C_5+_: 13.6%	^[^ ^150^ ^]^
Chemical synthesis					
Selective hydrogenation	Pd_1_/N‐G	Xe lamp	125	99% Acetylene to ethylene: 93.5%	^[^ ^72^ ^]^
	Pt–Fe/SiC	LED (400–800 nm, 0.4 kW m^−2^) and temperature controlling	20	100%/15 min 3‐Nitrostyrene to 3‐aminostyrene: 91.3%	^[^ ^151^ ^]^
Selective oxidation	SnO_2_:Sb	Xe lamp (>300 nm, 26 W m^−2^ at 320–400 nm)	/	Benzylamine to benzaldehyde: ≈90% / 24h	^[^ ^81^ ^]^
	ZnO@ZIF‐8	Xe lamp (3 kW m^−2^) and external heating	200	39.8% Ethanol to aldehyde: 91.5%	^[^ ^76^ ^]^
	MnO*_x_*/TiO_2_	Xe lamp (5.439 kW m^−2^)	206	59.1% Ethanol to aldehyde: 18.828 mmol g^−1^ h^−1^, 89.7%	^[^ ^152^ ^]^
	Pt/PCN‐224(M)	Xe lamp (>400 nm)	36	Aromatic alcohol to aldehyde: ≈100%/50 min	^[^ ^33^ ^]^
	WO_3_–Au	Xe lamp and external heating	120	9.0%/8 h CHA to KA oil^c)^: 99.0%	^[^ ^45^ ^]^
	WO_3_‐NCDs	Xe lamp and external heating	120	7.88%/8 h CHA to KA oil: 98.9%	^[^ ^153^ ^]^
	MoO_3_–Ag	Xe lamp and external heating	120	8.6%/8 h CHA to KA oil: 99.0%	^[^ ^53^ ^]^
	Au–Pt/Cu_7_S_4_–Cu_9_S_8_	Xe lamp (>400 nm)	50	Amine to imine: ≈100%/120 min	^[^ ^154^ ^]^
Coupling reaction	Cu_7_S_4_@ZIF‐8	laser (1450 nm, 500 mW)	94	Cyclocondensation: 97.2%/6 h	^[^ ^63^ ^]^
	M@CCOF‐CuTPP	Xe lamp (>400 nm, 25 kW m^−2^)	58	Asymmetric one‐pot Henry and A^3^‐coupling: TOF = 9.8 h^−1^ Enantiomeric excess: 98%	^[^ ^49^ ^]^
	Au–CuO	Xe lamp (420–780 nm) and external heating	60	1,3‐dipolar azide–alkyne cycloaddition: 90.6% / 2 h	^[^ ^56^ ^]^
	Pd–TiO_2_/CNF	Xe lamp and external heating	50	Suzuki coupling: 93.62% / 5 h selectivity: 94.80%	^[^ ^155^ ^]^
	Cu@Ni@ZIF‐8	Xe lamp	–	C–C coupling reaction of boric acid: 62%	^[^ ^79^ ^]^
Environmental remediation					
Gaseous contaminant treatment	CuO HCs	Xe lamp	≈200	CO: 99.3%/20 min, 482.1 μmol_CO_ g^−1^ h^−1^	^[^ ^62^ ^]^
	Fe_3_Si/Co_3_O_4_	Solar irradiation (0.3–0.35 kW m^−2^)	160	CO: >95%	^[^ ^112^ ^]^
	AlN*_x_* + W/Fe_2_O_3_	Solar irradiation (CR = 4)	270	NO*_x_* SCR: 90%	^[^ ^111^ ^]^
	TiO_2_(B)	Halogen lamp (365 nm, 10 W m^−2^) and external heating	60	NO*_x_* SCR: 70.01% Non‐NO_2_ selectivity: 93.73%	^[^ ^80^ ^]^
	Pt/TiO_2_–WO_3_	Xe lamp (with IR filter, 10 kW m^−2^) and external heating	90	C_3_H_8_: 70%	^[^ ^40^ ^]^
	Ag/Ag_3_PO_4_/CeO_2_	Xe lamp	135	Benzene: 90.18%/3 h; CO_2_: 46.72%; TOC: 74.17%	^[^ ^156^ ^]^
	Pt/TiO_2_(001)	Xe lamp (3.998 kW m^−2^)	209	Benzene: 45.195 mmol_CO2_ g^−1^ h^−1^	^[^ ^76^ ^]^
	Pt/*γ*‐Al_2_O_3_	Simulated sunlight (3.2 kW m^−2^)	169	Toluene: 94%/10 min	^[^ ^157^ ^]^
	CeO_2_/LaMnO_3_	IR lamp (2.8 kW m^−2^)	275	Toluene: 89%/120 min, 11.88 μmol_toluene_ g^−1^ h^−1^; CO_2_: 425.4 μmol g^−1^ h^−1^, 87%	^[^ ^158^ ^]^
	Pt/SrTiO_3‐_ *_x_*	Xe lamp (420–780 nm, 1.5 kW m^−2^) and external heating	150	Toluene: ≈ 100%/60 min	^[^ ^159^ ^]^
	A‐LaTi_1‐_ *_x_* Mn*_x_* O_3+_ *_*δ*_*	Xe lamp (6.5 kW m^−2^)	227.5	Toluene: 96%; CO_2_: 72%	^[^ ^52^ ^]^
	ARCeO_2_	Xe lamp (300–780 nm, 2 kW m^−2^) and external heating	226	Styrene: 90%	^[^ ^160^ ^]^
			495	*n*‐hexane: 90%	
			563	Cyclohexane: 90%	
Water treatment	MnO_2_‐G	Xe lamp	80	Formaldehyde: 87.2%/40 min; CO_2_: ≈100%	^[^ ^161^ ^]^
	GO/MnO*_x_*/CN	Xe lamp	≈85	Formaldehyde: > 90%/12 min	^[^ ^162^ ^]^
	Co*_x_* O/TiO_2_	LED (470 nm, 2 kW m^−2^) and external heating	60	Acetaldehyde: ≈100%	^[^ ^46^ ^]^
	H‐Ov‐TiO_2_(AB)	Xe lamp (350–400 nm, 30 W m^−2^) and external heating	70	Acetaldehyde: ≈100%/40 min	^[^ ^85^ ^]^
	Zn_x_Cd_1‐_ *_x_* S/Bi_2_S_3_	Xe lamp (15 kW m^−2^)	46.7	RhB^c)^: 100%/30 min	^[^ ^162^ ^]^
	C@TiO_2_	Xe lamp (>420 nm) and external heating	60	RhB: 92.7%/150 min	^[^ ^64^ ^]^
	Flower‐like CuS	Xe lamp (10 kW m^−2^)	≈65	MB^c)^: ≈100%/25 min	^[^ ^78^ ^]^
	Zr‐Fc MOF	Xe lamp (AM 1.5G, 1.0 kW m^−2^)	90	MB: > 99%/35 min integrated with water evaporation	^[^ ^121^ ^]^
	B‐TiO_2_	Xe lamp	78	MB: ≈100%/40 min	^[^ ^164^ ^]^
	Ag/TiO_2_	Xe lamp (>420 nm)	25	4‐NP^c)^: ≈ 100%/150 s	^[^ ^165^ ^]^
	Ag‐MBTH	Xe lamp (>420 nm, 1 kW m^−2^) and external heating	40	4‐NP:100%/26 s	^[^ ^166^ ^]^
	Ag/MoS_2_/TiO_2‐_ *_x_*	Xe lamp (>420 nm)	–	BPA^c)^: 96.7%/120 min	^[^ ^124^ ^]^
	Bi_5_O_7_I/Ag/CdS	Xe lamp (>420 nm)	Δ*T* ≈ 7	BPA: ≈97%/180 min	^[^ ^65^ ^]^
				2,6‐DCP^c)^: ≈93%/180 min	
	Ag/Bi_2_S_3_/MoS_2_	Xe lamp (>420 nm)	/	2,4‐DCP: 99.2%/210 min	^[^ ^67^ ^]^
	Cu_2‐_ *_x_* S/CdS/Bi_2_S_3_	Xe lamp (>420 nm)	/	2,4‐DCP: 99%/150 min	^[^ ^126^ ^]^
	*α*‐Fe_2_O_3_/MoS_2_/Ag	Xe lamp (>420 nm)	/	2,4‐DCP: ≈100%/120 min	^[^ ^66^ ^]^
		Xe lamp (420–780 nm)	/	Salicylic acid: 97%/135 min	
	AC/CN	Xe lamp	≈45	Sulfamerazine: 98%/60 min	^[^ ^167^ ^]^
		Solar irradiation (0.7 kW m^−2^)	35	Sulfamerazine: 99%/90 min	
	Bi‐BN/Ag–AgCl	Xe lamp	/	Ceftriatone sodium: 98.9%/210 min	^[^ ^168^ ^]^
			/	Cr(VI): 98.3%/210 min	

^a)^ The symbols of “/” in the Temperature column represent the unspecified reaction temperatures

^b)^ Necessary unit conversions have been made. In the “Solar Fuel Production” section, the percentages without additional information represent the conversion rates of the reactants, while the data of product selectivity are labeled with the product names and the data of energy efficiency are labeled with SE (solar efficiency)

^c)^ Compound abbreviations: CHA (cyclohexane), RhB (Rhodamine B), MB (methylene blue), NP (nitrophenol), BPA (bisphenol A), DCP (dichlorophenol).

When it comes to the popularization and application of a certain PTC reaction, priority should be given to the difficulty level of triggering the reaction and the development level of fundamental research. Most of uphill chemical reactions (especially the CO_2_ reduction with H_2_O) require a mass of energy input, usually with yields that are far from practical application. On the one hand, it is necessary to develop more elaborately designed experimental programs and more refined in situ characterization methods for deeper understanding of PTC mechanism and better design of effective catalyst. On the other hand, increasing the energy input by solar concentrator with high concentration ratio can promote the conversion rate and even obtain higher‐value products. As for the reactions with mature researches, it will be less challenging to push ahead with the engineering application. Thus, the system optimization and energy management would be more critical to achieve a higher overall efficiency. Here, we propose several key points which must be considered when pushing ahead with the engineering application of PTC.

#### 3.2.1 Suitable Concentrator Devices

**Figure 11:**
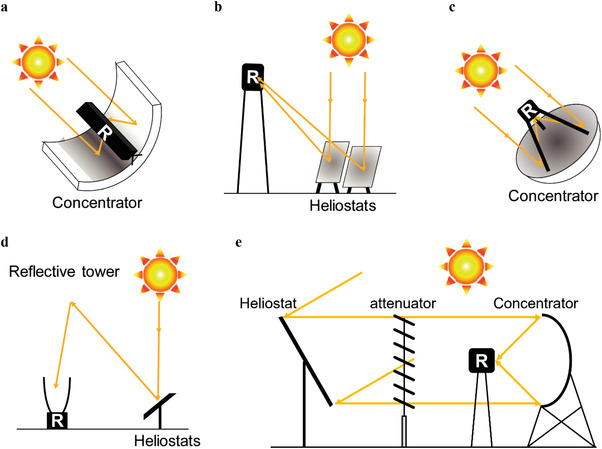
Main high temperature solar concentrator system categories: a) parabolic trough, b) central power tower, c) parabolic dish, and d) double concentration. Reproduced under the terms of the CC BY‐NC‐ND 4.0 license.^[^ ^14^ ^]^ Copyright 2017, Institute of Process Engineering, Chinese Academy of Sciences. Publishing services by Elsevier B.V. on behalf of KeAi Communications Co., Ltd. e) Solar concentrator system of the solar furnace SF40. Adapted with permission.^[^ ^108^ ^]^ Copyright 2016, AIP Publishing.

In laboratory, artificial light sources are usually applied for PTC researches (usually xenon lamp or halogen lamp for simulated solar irradiation, mercury lamp for UV irradiation, and LED for specific wavelength irradiation), because of the facile modulation of light intensity and spectrum and the illumination stability. However, in the practical applications, it is pointless to drive PTC processes by artificial light sources, since the utilization of sunlight is one of the original intentions of developing PTC. Up till now, there are few of outdoor experiments having been reported. As shown in Figure 10 and Table 1, the reaction temperatures of different PTC processes mainly range from room temperature to 800 °C, while the irradiation intensities are diverse as well. Except for some degradation reactions that can take place under AM 1.5 irradiation, most PTC reactions required a higher intensity of light than the sunlight received on Earth. According to the required irradiation intensity and the reaction temperature, a concentrator may be needed. There are several types of concentrators with different concentration ratios, as shown in **Figure** **11**. The concentration ratios can be attained in the range of 30–100 (for a parabolic trough system to achieve 250–450 °C temperatures) and up to 5000–10 000 (in a double‐concentration system consisting of a heliostat field, a reflective tower and a ground receiver, capable of temperatures beyond 1200 °C).^[^ ^22^ ^]^ Another kind of concentration system, which generally combines a heliostat, a concentrator and a cavity receiver–reactor, is also common in the representative solar furnaces built by international research institutions, as shown in Figure 11e.^[^ ^108^, ^109^ ^]^ The heliostat is used to track and reflect sunlight, while the concentrator, an integrated parabolic dish or a quasi‐parabolic dish composed of many separated plane mirrors, concentrates the collimated light into the reaction chamber, with a CR of over 5000.

It is also reasonable to choose a suitable reaction system according to the conditions that the concentrator can provide. Among these concentration systems, the parabolic trough system may be the most competitive in PTC application, because it is more flexible to expand the light absorption area and thus the system scale. Besides, the parabolic trough system can match with the reactors with tubular structure, which is also facile to conduct reaction processes continuously.

#### 3.2.2 Elaborately Designed Catalysts and Reactors for Large‐Scale Production

It is with no doubt that more efficient PTC catalysts are needed for engineering application. To this end, the materials and architectures for PTC must meet several requirements, which include, but not limited to, strong light absorption across the entire solar spectrum, enhanced charge carrier separation efficiency, and high capacity for heat generation.^[^ ^10^ ^]^ A number of PTC catalysts that have been synthesized and approved in previous studies involving different fields. There are also some reviews that have summarized and categorized the catalyst candidates, and proposed a number of catalyst design strategies to achieve better catalytic performance and higher energy efficiency.^[^ ^3^, ^10^, ^16^, ^18^, ^110^ ^]^ Moreover, other characteristics, such as sufficient raw materials, simple preparation, system compatibility, low cost, long lifetime, and environment‐friendly, are important factors to considerate when designing the PTC catalysts. From the perspective of engineering application, simply combining light‐absorbing materials and catalytically active materials is a convenient approach to obtain both heat collection capacity and catalytic activity.

**Figure 12:**
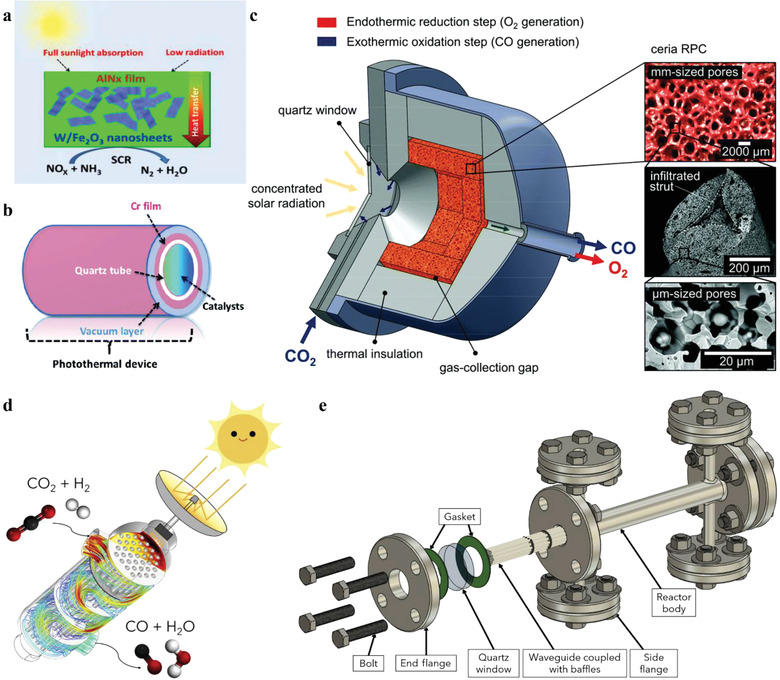
a) Schematic illustration of solar‐driven NO*_x_* SCR through W/Fe_2_O_3_ nanosheets equipped with AlN*_x_* film. Reproduced with permission.^[^ ^111^ ^]^ Copyright 2020, Elsevier B.V. b) Schematic of the photothermal device. Reproduced with permission.^[^ ^113^ ^]^ Copyright 2020, The Royal Society of Chemistry. c) Schematic of the solar reactor configuration for CO_2_ reduction via a two‐step thermochemical redox cycle. Reproduced with permission.^[^ ^114^ ^]^ Copyright 2017, The Royal Society of Chemistry. d) Schematic illustration of HI‐Light, a surface‐engineered glass‐waveguide‐based “shell‐and‐tube” type photothermal reactor. e) The assembly view of the reactor. Reproduced with permission.^[^ ^115^ ^]^ Copyright 2020, The Authors. Published by Elsevier.

Adapted from the vacuum tube collectors, tubular reactor can easily implement this approach. Bai et al. selected an AlN*_x_* film, which has been applied in commercial solar water heaters, to assist selective catalytic reduction of nitrogen oxides (NO*_x_* SCR) over W‐doped Fe_2_O_3_ nanosheets with a quadruple focusing parabolic reflector, as shown in **Figure** **12**a.^[^ ^111^ ^]^ The catalysts could be heated to 270 °C, attaining a NO*_x_* conversion of nearly 100% under outdoor irradiation. Besides, Lou et al. used Fe_3_Si aerogel to absorb sunlight, while Shi et al. used a chromium film.^[^ ^112^, ^113^ ^]^ A layer of Fe_3_Si was coated on an aluminized glass tube surface and made into an evacuated tube collector, which has both excellent solar‐to‐heat conversion efficiency and thermal storage capacity. This device was able to achieve over 150 °C under 0.3 kW m^−2^ of solar irradiation and excite the activity of Co_3_O_4_ rhombus‐shaped nanorods for complete CO oxidation inside the tube.^[^ ^112^ ^]^ Figure 12b shows the photothermal device based on a chromium film, which could heat CuO*_x_*/ZnO/Al_2_O_3_ nanosheets to around 300 °C under one standard solar irradiation and generate hydrogen from efficient water‐gas shift reaction (WGSR).^[^ ^113^ ^]^ In these cases, the solar‐to‐heat conversion is conducted by specialized materials and the heat is transferred to the reaction sites. Since the absorption and catalytic properties are separated, the design of materials and reactor systems is simplified. In addition to the tubular reactors applied with linear concentrators, cavity reactors also play an important part, especially in large‐scale solar furnaces, which can achieve higher temperature with double‐concentration system. As shown in Figure 12c, the reaction chamber is usually loaded with porous or arrayed catalyst materials, exposed to the concentrated solar radiation that passes through the quartz window.^[^ ^114^ ^]^ The thermal gradient can be very high in this kind of system, which is favorable for some reaction processes.

When the non‐thermal effect is needed, the PTC systems will become more complicated because of the imperative direct exposure of the catalysts to irradiation. In this case, cavity reactors have advantages over aforementioned tubular reactors, since they can meet the requirement of direct illumination. However, the direct illumination area is insufficient in the cavity reactors. It is better to assemble these two functions into one material and improve the reactor structure design. To enable uniform light distribution over all the catalyst surface and make it scalable in the meantime, Cao et al. have designed HI‐Light, a surface‐engineered glass‐waveguide‐based “shell‐and‐tube” type photothermal reactor, as shown in Figure 12d,e.^[^ ^115^ ^]^ It can provide efficient light coupling for photocatalytic reactions at elevated temperatures and is scalable in diameter and length.

#### 3.2.3 Multi‐System Integration

In order to study the mechanism of PTC, external heating methods are commonly used to assist PTC experiments in academic studies. With external heating, it is facile to attain the required temperatures and control the variables precisely. In practical applications aimed at making full use of solar energy, the ideal scenario is that both thermal and non‐thermal effects come from solar irradiation, which can exactly meet the requirements of PTC processes. However, this ideal scenario is quite difficult to achieve, as the spectral distribution of the solar spectrum is definite and the thermal and non‐thermal effects might not be optimal for the reaction systems. By designing the catalysts and device structures elaborately, these two effects can be modulated, yet which is a complicated and arduous task. The more feasible solution is to integrate other complementary processes into PTC system, with a key idea that photons with different energy levels are allowed to play different roles.^[^ ^116^, ^117^, ^118^, ^119^, ^120^ ^]^ Generally, the photons with higher energy are used to drive chemical reactions via non‐thermal effect, while those with lower energy go through the process of light‐to‐heat conversion, which can be used for thermochemistry, water heating, or other thermal utilizations. In integrated systems, the contributions of thermal effect and non‐thermal effect are easier to be regulated than in a single PTC system. The redundant heat converted from the sunlight can be exported for other utilizations, or the waste heat from some other processes can be brought in when heat is insufficient, increasing the efficiency integrally.

**Figure 13:**
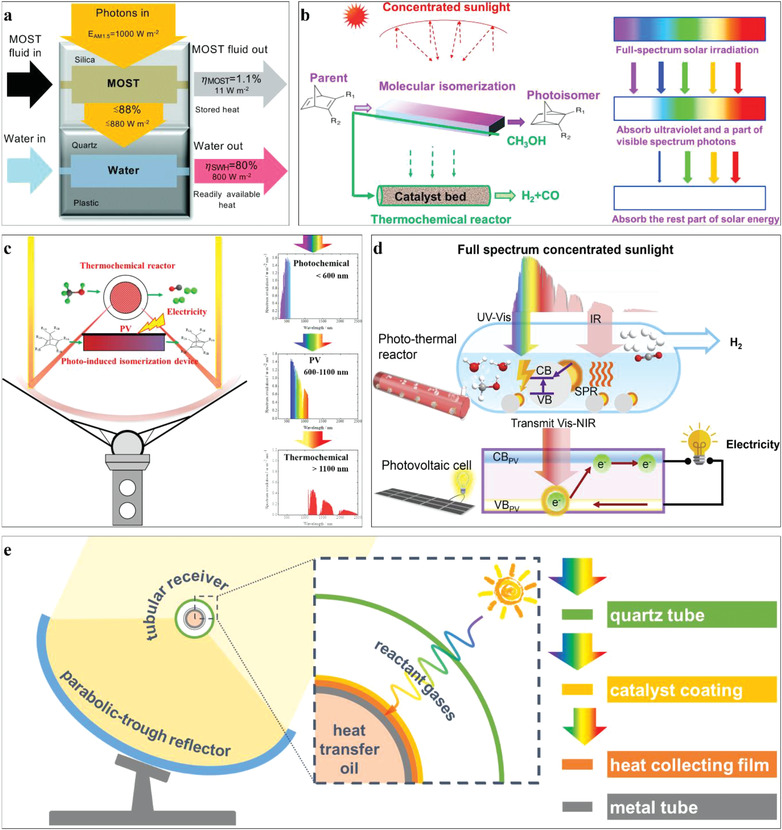
a) Sketch of the hybrid solar energy conversion device: the upper collector is used for the conversion of the MOST system; the lower collector is used for solar water heating. Reproduced with permission.^[^ ^116^ ^]^ Copyright 2017, The Royal Society of Chemistry. b) Mechanism of storage of the full spectrum of solar energy as chemical energy. Reproduced with permission.^[^ ^117^ ^]^ Copyright 2019, Elsevier Ltd. c) Schematic diagram of full‐spectrum solar energy utilization system and the cascade utilization of sunlight. Adapted with permission.^[^ ^118^ ^]^ Copyright 2020, Elsevier Ltd. d) Schematic of the concentrated solar system synergizing photothermal H_2_ and PV electricity in a cascade pathway. Reproduced with permission.^[^ ^120^ ^]^ Copyright 2020, The Author. Published by Elsevier. e) Schematic of the integrated system coupling PTC with heat collection.

To attain higher efficiency of solar energy conversion, researchers have tried to integrate diverse processes. Due to the thermal effect in PTC, water evaporation is facile to couple with water treatment and other photodriven processes.^[^ ^121^ ^]^ Dreos et al. have proposed a hybrid solar energy system, where a molecular solar thermal (MOST) energy storage system was integrated with a solar water heating system (SWH).^[^ ^116^ ^]^ As shown in **Figure** **13**a, the MOST layer is on the top of the SWH layer. The high‐energy photons from the solar spectrum can be absorbed by the upper MOST layer, and photochemically convert norbornadiene to quadricyclane, storing solar energy in the form of chemical energy at around 103 kJ mol^−1^. In the meantime, the low‐energy photons, accounting for ≈88% in the solar spectrum, will be absorbed by the lower SWH layer to heat the water. The efficiencies can achieve 1.1% and 80% for the MOST part and the SWH part, respectively.

Liu and co‐workers have integrated a photochemical process with a thermochemical process to convert the full spectrum of solar energy into chemical energy (Figure 13b).^[^ ^117^ ^]^ In the photochemical device, the ultraviolet–visible light photons are absorbed to drive the isomerization of norbornadiene derivatives; while the rest of photons corresponding to the visible‐infrared spectrum are transmitted to thermochemical reactors, providing heat for methanol decomposition. Under the design condition, the average solar chemical efficiency can reach 75.38%, which is higher than that of a single photochemical system or a single thermochemical system. Later on, they further integrated PV cells into the dual chemical energy storage system, as shown in Figure 13c.^[^ ^118^ ^]^ On the one hand, the photons with much higher energy than the *E*_g_ of photovoltaic cells are used in the photochemical process, decreasing the irreversible losses of high‐energy photons. On the other hand, the photons with lower energy than the *E*_g_ are recycled to provide heat for the thermochemical process. Compared with common concentrated photovoltaic‐thermal systems, the solar utilization efficiency of high‐energy photons (<600 nm) was increased from 44.01% to 80.68%. In this concentrated photochemical–photovoltaic–thermochemical (CP–PV–T) system, cascade utilization of full‐spectrum solar radiation was realized with a total solar utilization efficiency of 66.95% under the design condition.

Due to the temperature elevation under the concentrated solar irradiation, it is more practical to couple with PTC processes than to couple with pure photochemical processes. As shown in Figure 13d, Tang et al. reported a hybrid system synergizing photo‐thermochemical hydrogen and photovoltaics.^[^ ^120^ ^]^ A simple suspension of Au–TiO_2_ in water/methanol served as a spectrum selector, absorbing ultraviolet and part of visible‐infrared energy, while the rest of visible and near‐infrared light was coincided with the PV bandgap. In this way, the heat relaxation and recombination of PV are reduced, thus retaining high efficiency under concentrated sunlight. An overall efficiency of 4.2% was obtained at 12 suns, higher than the sum of efficiencies of individual a single PV system and a single PTC system.

Since solar thermal power generation is a mature technology and has been widely applied, modifying solar thermal power generation devices for PTC processes is a feasible solution, which can integrate with heat collection and meet the temperature requirement for the PTC processes in the meantime. In our recent work, a system that couples gas‐phase PTC with heat collection has been established, adapted from parabolic trough solar thermal power generation device.^[^ ^122^ ^]^ As shown in Figure 13e, the integrated system contains a tubular receiver and a parabolic‐trough reflector. The receiver consists of two concentric tubes, which acts as both a heat collector and a PTC reactor. The exterior of the inner tube is coated with a layer of heat collecting film and a thin layer of catalyst. With the development of materials, heat collection and catalysis can be achieved on one material. Inside the inner tube, heat transfer oil is circulated to collect and transfer heat, while the space between the inner tube and the glass envelope is filled with reactant gas. When concentrated sunlight irradiates the receiver, ultraviolet and part of visible light is absorbed by the catalyst to activate the reaction, while the rest of light is absorbed by the heat collecting film. The surface of catalyst coating can be heated to 300–400 °C under concentrated solar radiation. A part of generated heat promotes the PTC reactions, while the rest is transferred through the wall and stored in the oil. In this way, the photons with higher energy can drive the chemical reactions, while the rest of energy can be collected in the form of heat, enabling the cascade utilization of full‐spectrum solar radiation.

## 4 Conclusion and Outlook

With energy and environmental problems becoming increasingly prominent, driving chemical reactions by solar energy is an attractive solution. Compared with the low spectral efficiency and low reaction rate of photochemistry, PTC takes advantage of visible and infrared light, which widens the range of spectral absorption and promotes the reaction rate. PTC‐T, which involves thermal effect only, is a promising alternative to the traditional heating methods. When the thermal and non‐thermal effects synergize, PTC‐S can obtain a higher reaction rate under relatively mild conditions and achieve full‐spectral utilization of solar radiation.

Earlier in the article, we introduced the PTC mechanisms in detail from two aspects: the non‐thermal effect and the thermal effect. After summarizing their synergistic performances and some adverse scenarios, a better understanding of PTC has taken shape. Later, focusing on distinguishing and quantifying these two effects, we reviewed the experimental approaches and characterization methods in recent studies to guide effective PTC catalyst design and ascertain the optimal reaction conditions. In addition to laboratory studies, the engineering application of PTC is the ultimate aim. After developing more elaborately designed experimental programs and more detailed in situ characterization methods for deeper understanding of the PTC mechanisms and better design of effective catalysts, several key points which must be considered when pushing ahead with the engineering application of PTC.

- When shifting artificial light sources to solar irradiation, suitable concentrator systems must be considered in order to obtain the required irradiation intensity and temperature.
- PTC reactors can be adapted from existing solar devices by simply combining light‐absorbing materials and catalytically active materials. However, there are still some special requirements that need to be met in practical application.
- Furthermore, integrating different systems is a feasible way to attain cascade utilization of full‐spectrum solar radiation.

With the development of catalyst design, mechanism research and system integration, PTC based on solar energy would become more feasible for practical applications, and full‐spectral utilization of solar radiation would be achieved.

## Conflict of Interest

The authors declare no conflict of interest.
